# Nucleic acid vaccination strategies for ovarian cancer

**DOI:** 10.3389/fbioe.2022.953887

**Published:** 2022-11-07

**Authors:** Chayanika Saha, James Bojdo, Nicholas J. Dunne, Raj Kumar Duary, Niamh Buckley, Helen O. McCarthy

**Affiliations:** ^1^ School of Pharmacy, Queen’s University of Belfast, Belfast, United Kingdom; ^2^ School of Mechanical and Manufacturing Engineering, Dublin City University, Dublin, Ireland; ^3^ Centre for Medical Engineering Research, School of Mechanical and Manufacturing Engineering, Dublin City University, Dublin, Ireland; ^4^ Department of Mechanical and Manufacturing Engineering, School of Engineering, Trinity College Dublin, Dublin, Ireland; ^5^ Advanced Manufacturing Research Centre (I-Form), School of Mechanical and Manufacturing Engineering, Dublin City University, Dublin, Ireland; ^6^ Advanced Materials and Bioengineering Research Centre (AMBER), Royal College of Surgeons in Ireland and Trinity College Dublin, Dublin, Ireland; ^7^ Advanced Processing Technology Research Centre, Dublin City University, Dublin, Ireland; ^8^ Trinity Centre for Biomedical Engineering, Trinity Biomedical Sciences Institute, Trinity College Dublin, Dublin, Ireland; ^9^ Department of Food Engineering and Technology, Tezpur University, Tezpur, India; ^10^ School of Chemical Sciences, Dublin City University, Dublin, Ireland

**Keywords:** ovarian cancer, high grade serous carcinoma, tumour antigens, nucleic acid vaccines, DNA, mRNA

## Abstract

High grade serous carcinoma (HGSC) is one of the most lethal ovarian cancers that is characterised by asymptomatic tumour growth, insufficient knowledge of malignant cell origin and sub-optimal detection. HGSC has been recently shown to originate in the fallopian tube and not in the ovaries. Conventional treatments such as chemotherapy and surgery depend upon the stage of the disease and have resulted in higher rates of relapse. Hence, there is a need for alternative treatments. Differential antigen expression levels have been utilised for early detection of the cancer and could be employed in vaccination strategies using nucleic acids. In this review the different vaccination strategies in Ovarian cancer are discussed and reviewed. Nucleic acid vaccination strategies have been proven to produce a higher CD8^+^ CTL response alongside CD4^+^ T-cell response when compared to other vaccination strategies and thus provide a good arena for antitumour immune therapy. DNA and mRNA need to be delivered into the intracellular matrix. To overcome ineffective naked delivery of the nucleic acid cargo, a suitable delivery system is required. This review also considers the suitability of cell penetrating peptides as a tool for nucleic acid vaccine delivery in ovarian cancer.

## 1 Introduction

Ovarian Cancer (OC) is a silent gynaecological cancer with approximately 820,000 cases worldwide and _∼_4,000 deaths per year in 2020 in the United Kingdom alone (GLOBOCAN, 2020- International Agency for Research on Cancer). Currently, the five-year survival rate for OC is a mere _∼_30% for patients with advanced disease and this mortality is attributed to delayed diagnosis, relapse and resistance to standard of care therapies. (Xie et al., 2019) Existing key screening techniques are ineffective to diagnose the disease at an early stage owing to the heterogenous nature of the tumours.

**FIGURE 1:**
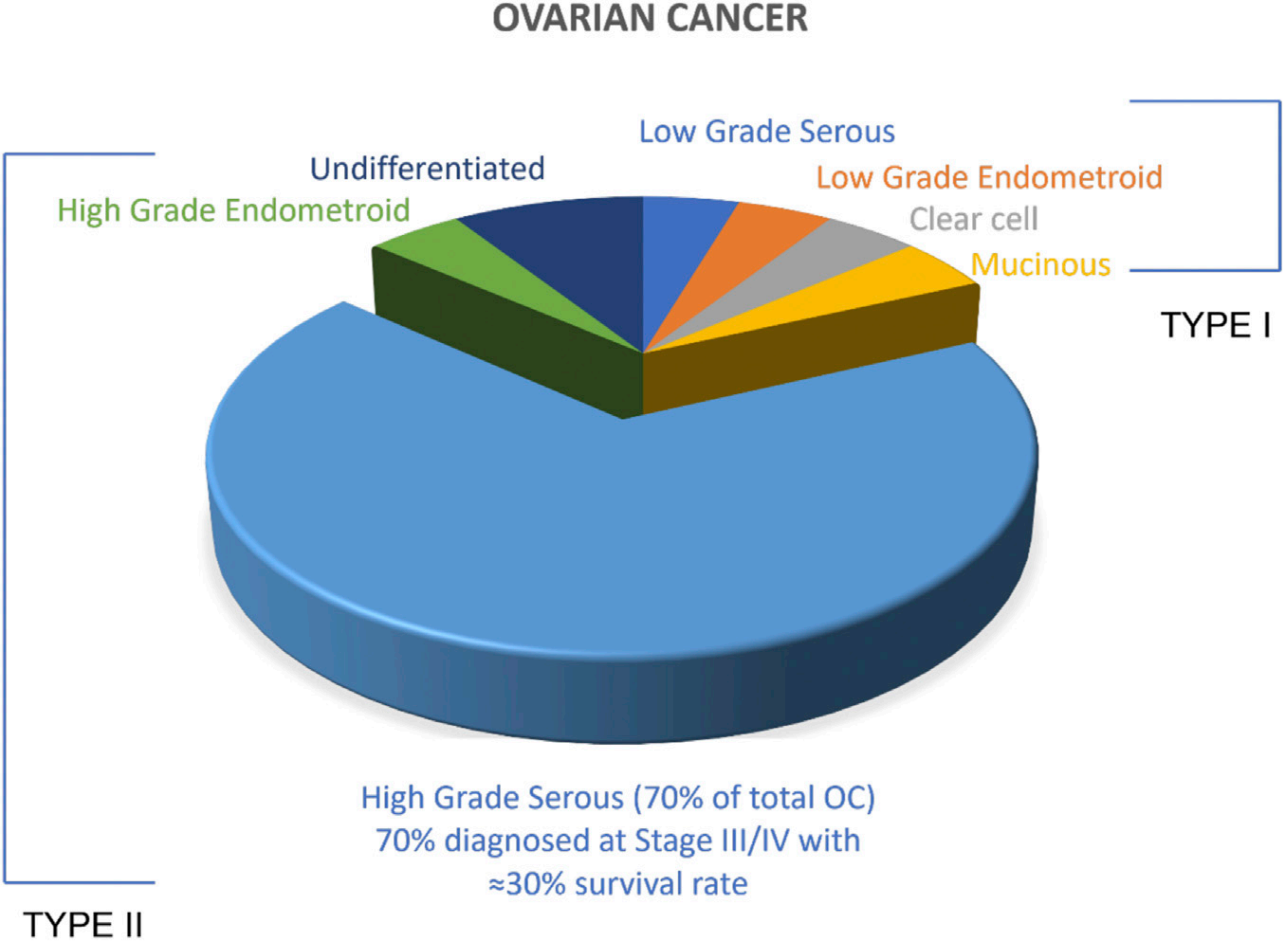
Ovarian Cancer types. All tumours associated with OC have been broadly classified into two subtypes: Type I and Type II. Malignant Type II comprises >85% of the total OC.

**FIGURE 2:**
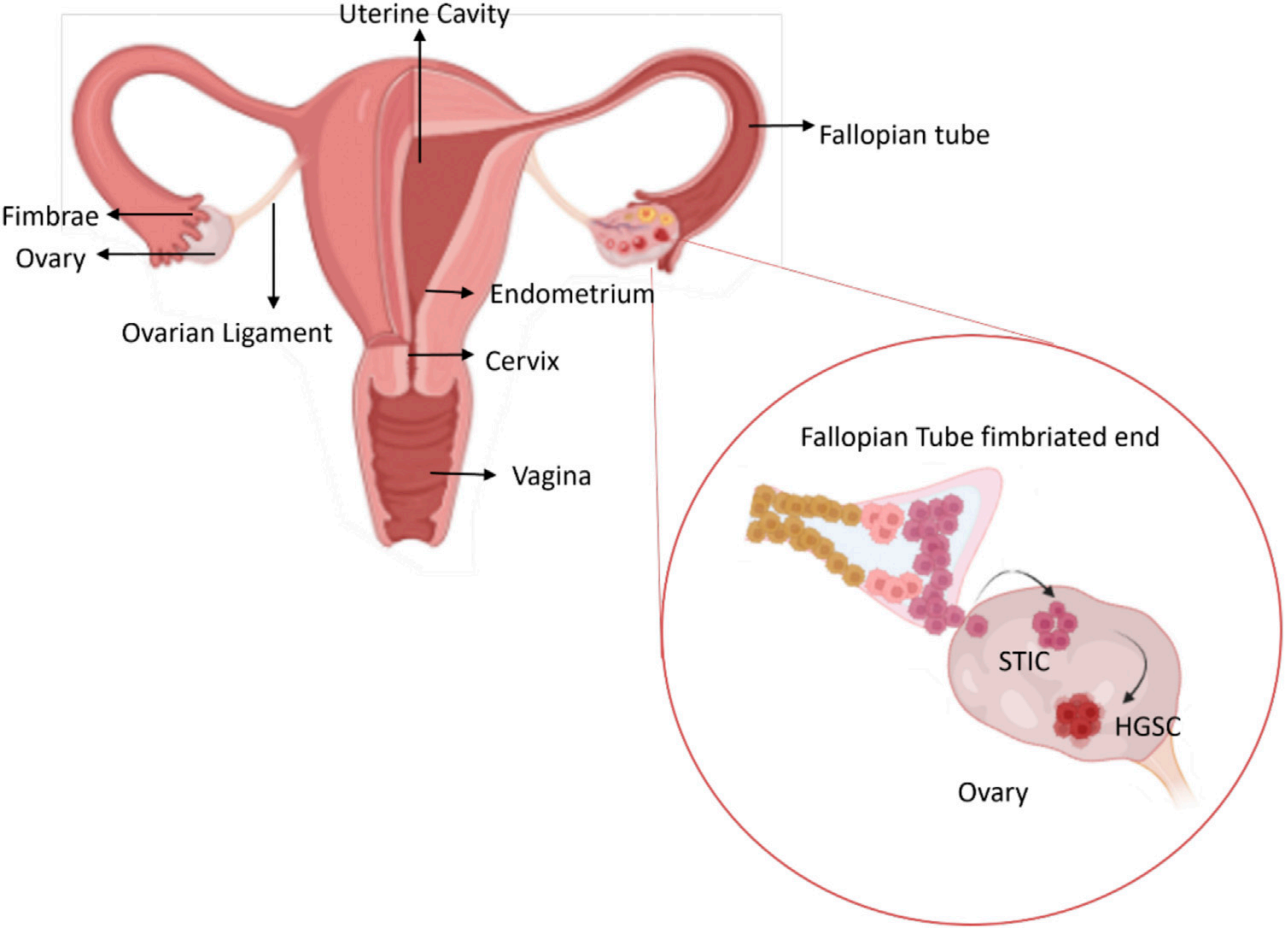
The origin of HGSC OC, most common form of EOC. A precursor lesion known as serous tubal intraepithelial carcinoma (STIC) in the fallopian tube are considered as the site for the origin of the malignant cells and involve the ovaries only secondarily (Created in BioRender.com).

There are several OC tumour subtypes which are categorised as either Type I or Type II based on the cells of origin of the tumour (Figure 1). Type I is mostly benign and shares lineage with the cystic neoplasms and corresponding carcinomas such as endometriomas, cystadenomas. Type I OCs include low-grade endometroid, low-grade serous clear cell and mucinous carcinomas. Type II are the more aggressive OC, evolve more rapidly as a result of genetic instability, and are mostly malignant in nature. Type II tumours include high grade serous carcinoma (HGSC), high-grade endometroid, undifferentiated carcinoma and carcinosarcoma HGSC is the most prevalent of the OC subtypes accounting for approximately 70% of cases. Clinically, HGSC is asymptomatic, which delays diagnosis and this results in high rates of fatality. (Kurman and Shih, 2011; Beirne et al., 2019; Ahmed et al., 2020) The treatment of HGSC has been stagnated because of the lack of research and understanding about the origin of HGSC. Recently, HGSC cells were identified to originate from the fallopian tubes rather than the ovary (Figure 2). Robert et al. proposed a unifying theory for the origin of HGSC suggesting serous tubal intraepithelial carcinoma (STIC), a precursor lesion in the distal, fimbrial end of fallopian tubes, were the cells of origin for HGSC with the involvement of the ovaries only at a secondary stage. (Kurman and Shih, 2010) Immunohistochemical experiments conducted by a group of researchers in the Netherlands provided an early linkage between the neoplastic lesions arising in the fallopian tube to the development of ovarian cancer. (Piek et al., 2001) A recent study on the molecular pathology by Crum et al. (2013) also imply fimbria as the site of origin for the cells of HGSC. This has been widely adopted as a survey conducted by McCluggage et al. (2017) predicts the wide spread acceptance of STIC being the origin of HGSC by 92% of clinicians and 86% of pathologists.

The primary clinical treatment option available for OC patients in the advanced stages is cytoreductive surgery followed by first line of therapy with three weekly intravenous injections of or platinum/taxane chemotherapy (paclitaxel, cisplatin, carboplatin). However the post-primary treatment rate of relapse is very high (>70%) in stage III/IV HGSC patients, which is the main contributor to the high mortality rate in women over 45 years old in Europe and Northern America. (Stuart, 2003) This highlights the urgent need for alternative treatment methods. Immunotherapy is a treatment option that has garnered interest. Immunotherapy can be described as programming the body to eliminate the cancer and this has developed significantly over the past 15 years. Cancer immunotherapy is a broad term to describe the method to induce, enhance or supress the immune response to tumour cells. Targeted therapies such as bevacizumab (VEGF), cetuximab (EGFR) and trastuzumab (HER2) have been used successfully as a combination therapy to combat OC. (Franzese et al., 2019) While, these alternative therapies offer more targeted treatment options with minimal cytotoxicity to the non-cancerous normal healthy cells, the success rate remains sub-optimal and there is a requirement for better therapies. This review examines the options for nucleic acid vaccines along with possible adjuvants that could be used for ovarian cancer.

## 2 Tumour antigens in ovarian cancer

Development of an immunotherapy requires a target that is either involved in immune tolerance of the cancer, is exclusively expressed, or is overexpressed by the cancer tissue. Tumour antigens (TAs) was coined for the proteins that have abnormal expression in the cancer tissue. (Wang and Wang, 2016; Odunsi, 2017a) TAs can either be mutational antigens/tumour specific antigens (TSAs) or non-mutated overexpressed tumour associated antigens (TAAs). Over the years researchers have measured the expression of different TAs in different OC patient tumour samples with the help of Deoxyribonucleic Acid/Ribonucleic Acid (DNA/RNA) sequencing, Reverse Transcriptase Polymerase Chain Reaction (RT PCR) and Western blotting techniques. Table 1 summarises a list of antigens that are differentially expressed in OC samples.

**TABLE 1:** Table of identified antigens in Ovarian cancer. These antigens are either mutated or overexpressed in the various types of the ovarian carcinomas and can serve as potential targets for immunotherapy against ovarian cancer cells. The blank spaces indicate that the significance and precise role of the genes in OC is not known and yet to be determined.

Type	Antigen name	Band location	Significances and functions involved in OC	Positive % in OC	References
TAA	ABCB1 (MDR1)	7q21.1	Negative influence on chemosensitivity	86% (177/206)	Lu et al. (2007); Vaidyanathan et al. (2016); Wu et al. (2016)
TAA	AKAP3	12p13.32	Increased motility, increased risk of metastasis and delayed prognosis	58% (43/74)	Hasegawa et al. (2004); Sharma et al. (2005a); Sharma et al. (2005b)
TAA	AKAP4	Xp11.22	Influences cell cycle, inhibits apoptosis and increased epithelial mesenchymal transition, increased metastasis	89% (34/38)	Agarwal et al. (2013); Kumar et al. (2017)
TAA	BAGE	21p11.2	Formation and development of ascites	14.6 % (6/41)	Gillespie et al. (1998); Zhang et al. (2010)
TAA	BIRC5	17q25.3	Inhibition of Apoptosis	60% (25/41)	Wang et al. (2018a); Trnski et al. (2019)
TAA	BORIS	20q13.31	DNA hypomethylation	—	Woloszynska-Read et al. (2007); Woloszynska-Read et al. (2011); Link et al. (2013)
TSA	BRCA 1	17q21.31	Influences DNA repair, cell cycle checkpoint regulation and transcription	100% (18% mutated 36/201)	Kurman and Shih. (2011); Wang et al. (2018b); Tsibulak et al. (2018)
TSA	BRCA 2	13q12.3	Influences DNA repair, cell cycle checkpoint regulation and transcription	100% (6% mutated 11/201)	Kurman and Shih. (2011); Wang et al. (2018b); Tsibulak et al. (2018)
TAA	CA125/MUC16	19p13.2	Indicates malignancy, promotes cell proliferation, inhibits anticancer innate immune response, influences cancer cell signalling, increases metastasis	90% (37/41)	Leung et al. (2013); Felder et al. (2014); Kloudová et al. (2016); Sallum et al. (2018); Verri et al. (2020)
TAA	CEACAM21	19q13.2	Promotes tumourigenesis by inhibition if cellular differentiation	—	Kreuzinger et al. (2017)
TAA	CT-45	Xq26.3	Influences DNA repair- damage response, increased Chemosensitivity	37% (82/219	Chen et al. (2009); Zhang et al. (2015); Coscia et al. (2018)
TAA	EPCAM	2p21	Enhances tumour initiating ability of ovarian stem like cells	90% (37/41)	Van Elssen et al. (2010); van der Gun et al. (2013); Kloudová et al. (2016)
TAA	FOLR1	11q13.4	Influences nucleic acid synthesis and cellular metabolism	90% (37/41)	Leung et al. (2013); Köbel et al. (2014); Kloudová et al. (2016)
TAA	GAGE-1/2	Xp11.23	—	14.65% (40/273)	Gillespie et al. (1998); Duan et al. (1999); Materna et al. (2007)
TAA	Her2/neu	17q12	Regulates cell proliferation, DNA damage, tumour cell metastasis	31.53% (35/111)	Verri et al. (2005); Demir et al. (2014); Wang et al. (2016); Verri et al. (2020)
TAA	HORMAD1	1q21.3	Influences angiogenesis, cell cycle and ascites	76.1 % (68/90)	Shahzad et al. (2013)
TAA	HSP70-2	6p21.33	Influences cell proliferation, colony forming abilities and cell viability	—	Gupta et al. (2017)
TAA	KIF2A	5q12.1	Influences cell migration and cell signalling	71.17% (79/111)	Verri et al. (2005); Wang et al. (2016); Sheng et al. (2018)
TAA	LAGE-1	Xq28	—	40% (42/107)	Odunsi et al. (2003); McCormack et al. (2013)
TAA	MAGE-A1	Xq28	Indicator of degree of malignancy and clinical stage, Critical to cell survival and tumorigenesis, transformation of stem cells, associated with poor prognosis	40.3% (25/62)	Daudi et al. (2014); Srdelić et al. (2019)
TAA	MAGE-A3	Xq28	Critical to cell survival and tumorigenesis, transformation of stem cells	36% (131/300)	Batchu et al. (2014); Daudi et al. (2014); Esfandiary and Ghafouri-Fard. (2015)
TAA	MAGE -A4	Xq28	Critical to cell survival and tumorigenesis, transformation of stem cells, Correlates to other MAGE expression	47% (186/399)	Yakirevich et al. (2003); Daudi et al. (2014); Srdelić et al. (2019)
TAA	MAGE-A10	Xq28	Critical to cell survival and tumorigenesis, transformation of stem cells, associated with poor prognosis	52% (204/395)	Daudi et al. (2014); Kloudová et al. (2016)
TAA	MAGE-C1	Xq27.2	Critical to cell survival and tumorigenesis, transformation of stem cells	16% (42/267)	Daudi et al. (2014); Kloudová et al. (2016)
TAA	MUC1	1q22	Has a role in intracellular cell signalling, cell adhesion and in forming protective mucous barriers on epithelial surfaces	90% (37/41)	Van Elssen et al. (2010); Kloudová et al. (2016)
TAA	NY-ESO-1	Xq28	Corelates to high nuclear grade	41% (410/1,002)	Odunsi et al. (2003); Yakirevich et al. (2003)
TAA	OY-TES-1	12p13.31	-	81 % (87/107)	Fan et al. (2015)
TSA	p53	17p13.1	Mutated tumour suppressor gene, regulates cell cycle, apoptosis, DNA repair and senescence	70.7% (29/41)	Kloudová et al. (2016); Silwal-Pandit et al. (2017); Carter et al. (2018); Sallum et al. (2018); Zhu et al. (2021)
TAA	PIWIL1	12q24.33	Important role in tumour stem cell maintenance and differentiation, effects Tumour progression	90% (18/20)	Chen et al. (2013)
TAA	PIWIL2	8p21.3		95% (19/20)	
TAA	PIWIL3	22q11.23			
TAA	PIWIL4	11q21			
TAA	POTEs	-	Correlates to increased stage and grade, Role in apoptosis, cytoskeletal function	32.5% (13/40)	Bera et al. (2006); Barger et al. (2018); Sharma et al. (2019)
TAA	PLU-1/JARID1B/KDM5B	1q32.1	Influences gene expression ad chromatin structure	71% (85/120)	Wang et al. (2015)
TAA	PRAME	22q11.22	Stimulates cytotoxic T lymphocytes linked to hypomethylation phenotype	60% (70/119)	Griffioen et al. (2006); Zhang et al. (2016); Pankov et al. (2017)
TAA	RRBP1	20p12.1	Related to FIGO stage, histological type and grade and lymph node metastasis, regulates RNA stability, attenuates ER stress to survive tumorigenesis	77 % (83/100)	Ma et al. (2019)
TAA	SPAG9	17q21	Hotspot for chromosomal aberration, influences cellular interaction	90% (18/20)	Garg et al. (2007)
TAA	Sp17	11q24.2	Correlated to chemoresistance, Immune suppression, cell migration and metastasis	43% (30/70)	Straughn et al. (2004); Brunette et al. (2018); Gao et al. (2018)
TAA	SSX-1	Xp11.22	May be linked with cell migration and metastasis	2.5% (3/120)	Türeci et al. (1998); Valmori et al. (2006); Godefroy et al. (2007); Smith and McNeel. (2010)
TAA	SSX-2	Xp11.22		10% (12/120)	Türeci et al. (1998); Valmori et al. (2006); Smith and McNeel. (2010)
TAA	SSX-4	Xp11.22		16% (19/120)	Türeci et al. (1998); Valmori et al. (2006); Smith and McNeel. (2010)
TAA	TPBG	6q14.1	Involved in cell adhesion, may act as an inhibitor for Wnt/Beta-catenin signalling Pathway	60% (25/41)	Kloudová et al. (2016)
TAA	TRAG3	Xq28	Linked to chemoresistance, malignant phenotype	83.8% (31/37)	Duan et al. (1999); Duan et al. (2003); Yao et al. (2004); Materna et al. (2007)
TAA	WT1	11p13	Linked to higher grade and stage, influences mutational changes, global demethylation, and histone deacetylation	82.9% (34/41)	Hylander et al. (2006); Stewart et al. (2008); Kloudová et al. (2016); Carter et al. (2018); Sallum et al. (2018)

Interestingly, over 95% of HGSC cases have multiple mutations in the TP53 gene, mostly in the DNA binding domain. The codons in which these oncogenic mutations are found in HGSC are called the “hotspot” mutations—R175, Y220, G245, R248 and R273. (Deniger et al., 2018) These mutations may be oncogenic due to the loss of wild type variant but much is not known or reported in case of OC. The wild type p53 impacts the cytolytic T-lymphocyte (CTL) response to tumour cells. Transporter associated with Antigen Processing 1 (TAP 1) and Endoplasmic Reticulum Amino Peptidase 1 (ERAP1), miRNA34 and Fas/APO-1 an apoptosis inducing cell surface protein are upregulated in the wild type p53 which are directly involved in upregulation of antigen presentation through the molecular histocompatibility complex I (MHC I) pathway, degradation of Programmed death ligand -1 (PD-L1) transcripts-negative regulators of CTL activation and induction of apoptosis by the CTL after recognition of the antigen on the MHC I. Mutations in the wild type variant lowers the levels of TAP1, ERAP1, miRNA 34 and Fas/APO-1 and thus reduces the interaction between the MHC I on the cancer cells and T-cell receptors (TCR) on the CTLs thereby supressing the recruitment of CD8^+^ T cells and helping in the growth of the tumours. (Braun and Iwakuma, 2016) The germline mutations of the TSA BRCA1/2 also increase the chances of developing HGSC. BRCA1 and BRCA2 genes play important role in the homologous recombination repair of double strand break of DNA. (Neff et al., 2017) Antoniou et al. (2003) compared the data from four different studies conducted in the United States, Canada, Israel and Poland for ovarian cancer and found the average cumulative risks of developing ovarian cancer by age of 70 years was almost 39% of BRCA1 mutation carriers and 11% of BRCA2 mutation carriers. Other commonly reported TAAs which are over-expressed in ovarian cancer and can serve as potential biomarkers for immunotherapy include Cancer Antigen 125 (CA125) or Mucin 16 (MUC 16), (Leung et al., 2013; Felder et al., 2014; Kloudová et al., 2016; Sallum et al., 2018; Verri et al., 2020) Melanoma Antigen Gene - MAGEA1, MAGEA3, MAGEA4, MAGEC1, MAGEA10 protein family, (Gillespie et al., 1998; Yakirevich et al., 2003; Zhang et al., 2010; Batchu et al., 2014; Daudi et al., 2014; Esfandiary and Ghafouri-Fard, 2015; Srdelić et al., 2019) New York Esophageal squamous cell carcinoma-1 (NY-ESO-1), (Odunsi et al., 2003; Yakirevich et al., 2003) Cancer Testis antigen-45 (CT45), (Chen et al., 2009; Zhang et al., 2015; Coscia et al., 2018; Xie et al., 2019) Folate Receptor alpha (FOLR1), (Leung et al., 2013; Köbel et al., 2014; Kloudová et al., 2016) Epithelial Cellular Adhesion Molecule (EP-CAM), (Van Elssen et al., 2010; van der Gun et al., 2013; Kloudová et al., 2016) Erythroblastic Oncogene B (ERBB) also frequently called Human Epidermal growth factor Receptor 2 (HER-2/neu), (Demir et al., 2014; Wang et al., 2016; Verri et al., 2020) Wilm’s Tumour suppressor gene 1 (WT1), (Hylander et al., 2006; Stewart et al., 2008; Kloudová et al., 2016; Carter et al., 2018; Sallum et al., 2018) Baculoviral IAP repeat containing protein 5 (BIRC-5), (Wang et al., 2018a; Trnski et al., 2019) Preferentially expressed antigen in melanoma (PRAME), (Griffioen et al., 2006; Zhang et al., 2016; Pankov et al., 2017) Trophoblast glycoprotein (TPBG) (Kloudová et al., 2016) and Mucin 1 (MUC-1). (Van Elssen et al., 2010; Kloudová et al., 2016) The expression of Prostate, Ovary, Testes Expressed ankyrin domain family (POTE gene family) in EOC and HGSC has also been studied. POTEs expression in elevated levels was reported for few of the members of the family- POTEs (C, E, F and I) in HGSC. (Bera et al., 2006; Barger et al., 2018; Sharma et al., 2019).

Among the different TAAs the ones belonging to the subset of Cancer testis antigens (CTA) have been explored as potential biomarkers and candidates for vaccine strategies. CTAs are often members of multigene family encoded by ≈ 140 genes and mapped often on the X-chromosome (CTA-X) e.g.,- MAGE, CT45, NY-ESO1 etc., However, some CTA can be mapped in non-X chromosomes as well (non-CTA-X)—e.g., PRAME, BIRC5, BAGE etc., These are mainly expressed in the germ cells of the testes with little or negligible expression in normal healthy cells. (Odunsi, 2017b) CTAs have desirable immunogenicity with abhorrent higher frequency of expression in cancer cells due to DNA methylation or modification within the chromatin network. (Xie et al., 2019) Expression of CTAs is known to be restricted in the sites with privileged immunity such as the testes, fetal ovary and placenta and not in other normal healthy cells which provides a high immunogenicity when these genes are abnormally expressed in tumour cells. They also play an important role in tumour progression (soma-to germline transformation) and as such are the closest match compared to other TAs, for an ideal target for antigen specific immunotherapeutic response.

## 3 Cancer immune therapy

**FIGURE 3:**
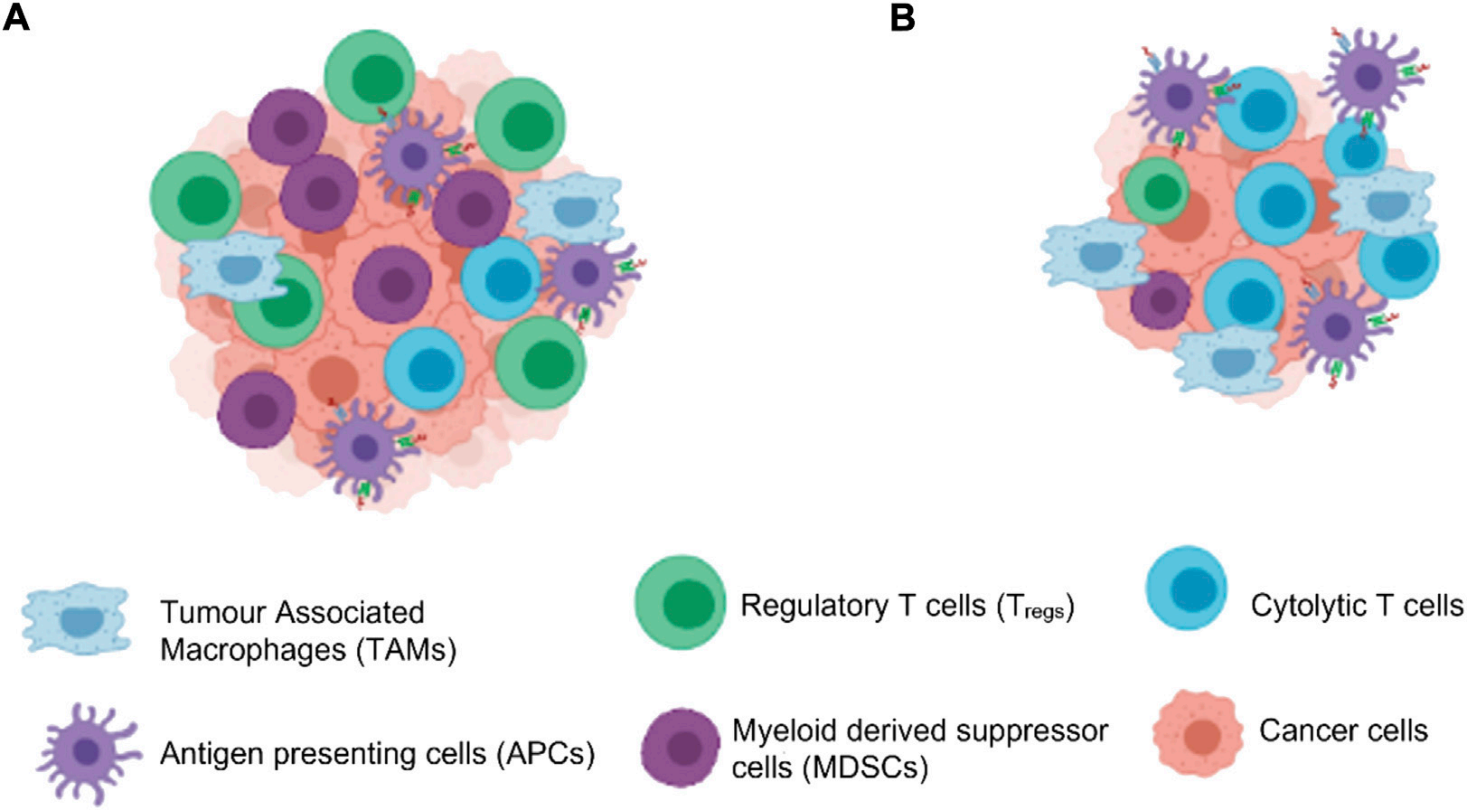
Cells in the tumour microenvironment. **(A)** High number of T_regs_ and MDSCs help in proliferation of the tumour cells evading the immune surveillance **(B)** Use of immunotherapy can increase the CTL in the tumour microenvironment thereby leading to decrease in the tumour growth by cell apoptosis. (Created with BioRender.com).

The immune system is a highly coordinated and complex process that involves interplay between the adaptive and innate immune system, which function to eliminate any foreign antigen that comes in contact with the body and repair damaged tissue or cells. In normal physiological conditions innate immune cells - the dendritic cells (DCs), natural killer cells (NKs), macrophages, neutrophils, basophils, eosinophils, and mast cells, are the first response generated against any foreign antigen during acute inflammation. The innate cells then activate the adaptive immune response by releasing mediators such as cytokines, chemokines and histamines which then retaliates to eliminate any damaged cells, extracellular matrix and invading pathogens.88 However, when tissue homeostasis is perturbed, the balance between both innate and adaptive immune system is lost and initiation of irreparable cell cycles are observed due to the improper engagement and disengagement of the two immune system arms. When a tumour develops in the body it tissue microenvironment consists of the tumour cells, blood vessels, stroma and infiltrating inflammatory immune cells. The host’s immune system tries to get rid of the cells through process of immune surveillance where immune infiltrates attempt to recognise and eliminate the tumour cells. These infiltrates can either be effector innate immune cells such as NK cells and macrophages as well as cells mediating adaptive immunity T-cells, B-cells and DCs. The immune landscape is however, characteristically altered within tumours with an imbalance of the tumour infiltrating lymphocytes (TILs) which are a major part of the tumour infiltrate which help them to evade immune surveillance. These TILs often have impaired T-cell receptor activity and therefore reduced cytotoxic activity and decreased production of cytokines to induce a T-helper cells Th1 stimulation against antigens. A skewed balance of T-helper Th1 cells towards Th2 in the tumour microenvironment is often seen in most tumours during progression. This imbalance dampens the cytolytic activity of the CD8^+^ CTLs and thus may help tumour growth. Activation of the nuclear factor -κB (NF-κB- a transcription factor) pathway in TILs increases the production of TNF-α as well as other proinflammatory cytokines responsible for cell proliferation in tumour cells. The most common TILs within a tumour are of regulatory (T_regs_) or myeloid derived suppressor cells (MDSCs) phenotype which actually aid with immune evasion and promote tumour growth (Figure 3). In normal conditions T_regs_ help to downregulate the immune system to reduce the risk of autoimmunity but in cancer when their number increases in the tumour microenvironment, they downregulate the functions of effector CD8^+^ CTLs and CD4^+^ CD25^−^ T-cells by secretion of inhibitory compounds TGF-β and IL10 and thus help in tumour growth. MDSCs also secrete TGF-β and accumulated levels in the tumour microenvironment which also dampens T-cell responses thus favouring tumour progression. MDSCs in most tumours also produces iNOS along with arginase one enzyme, which dampens the T lymphocyte response by increase in the production of superoxide and Nitric oxide (NO). Another enzyme, indoleamine-2,3-dioxygenase (IDO), produced by MDSCs catabolises tryptophan, which is an essential amino acid for T-cell proliferation and differentiation. Maturation defects in DCs in cancer patients increases the production of vascular endothelial growth factor (VEGF) in the tumours which help in the growth of blood vessels in the tumour aiding to its growth and metastasis. MDSCs are also recruited in the tumour by Granulocyte macrophage colony stimulating factor (GM-CSF) secreted by most tumour cells and thus induces immune suppression and tumour growth. (de Visser et al., 2006) GMCSF in normal physiological conditions help in maintaining homeostasis of immune cells but in tumour cells its presence may be impaired and may promote immune escape. The TILs are reprogrammed to continuously produce these growth factors which benefit the cell differentiation, blood vessel growth and benefit the tumour progression.

Immunotherapy manipulates the complex interaction between the immune cells, cytokines/chemokines and costimulatory substances increasing the immune surveillance to target cancer cells. Better understanding of the tumour tissue environment provides improved strategies to improve the antitumour effects. Higher migration and recruitment of effector CD8^+^ CTLs, blocking of MDSC activities and other proinflammatory compounds may provide some ideal arenas for antitumour therapies. Immunotherapies in cancer can thus be categorised as 1) immune checkpoint inhibitors with specific antibodies, (Lee and Konstantinopoulos, 2019) 2) monoclonal antibodies against specific tumour antigens, (Hamanishi et al., 2015) 3) adoptive T-cell therapy involving reinfusion of modified autologous T cells, (Tanyi et al., 2018) 4) naturally occurring or genetically modified oncolytic virus therapies, (Fukuhara et al., 2016) 5) Cancer vaccines. (Mittendorf et al., 2008; Mittendorf et al., 2014; Temizoz et al., 2016; Cole et al., 2018a; Kalli et al., 2018) Several recent reviews have discussed these different immune-oncological therapies in detail and so the scope of this review will focus specifically on the recent advancements and challenges of cancer vaccines along with future directions.

## 4 Cancer vaccines

Cancer vaccines are designed to programme the host’s immune system to detect and kill cancer cells predominantly by inducing a cellular immune response specific to TAA through the activation of APCs along with a lesser antibody response. At present, the vaccine platforms that are used for various trials in OC include - 1) autologous dendritic cell based 2) peptide or protein based, 3) nucleic-acid based.

### 4.1 The mechanism of cancer vaccines

DCs are considered as the most potent APC among the other immune cells. These cells can be either steady state conventional DC (cDCs—cDC1, cDC2) or non-conventional DCs arising from an inflammatory stimuli termed plasmacytoid DC (pDCs) or monocyte derived DC (MoDCs). The cDCs can further be classified into either migratory or lymphoid DCs. Surface markers on DCs are used to identify the different subsets DC. All DCs express the surface markers CD11c, CD45, MHC II and CD135. The cDC2 subset is CD4^+^ and expresses CD11b, CD80, CD86 and CD40 but do not express CD8 and CD205 otherwise expressed by cDC1. Unlike lymphoid cDC1 the non-lymphoid migratory cDC1 express CD103 instead of the CD8 and do not express CD11b, SIRPα(CD172A), F4/80 or CD115 which helps to differentiate them from macrophages, cDC2 and monocytes.

APCs take up the foreign antigen and display it in on the MHC I or II pathway depending upon whether the antigen is endogenous or exogenous. In a peptide or protein form the antigens are cleaved in the Endoplasmic Reticulum (ER) and directly represented on the MHC II molecules whereas if the antigens are in nucleic acid form (DNA/RNA) they are further processed and translated into protein inside the cells and then cleaved before presented in the MHC I complex. The activated DCs then migrates to the lymphoid organs such as lymph nodes, thymus, spleen etc., to activate and prime the naïve T cells—CD4^+^ or CD8^+^ cells into effector T cells or memory cells.88 Compared to cDC2, cDC1 have the higher ability to cross present antigens and activate a CD8^+^ cytolytic T cell response. The CD4^+^ cells detect antigens on the MHC II molecules and differentiate into T helper cells (Th1, Th2, Th17) generating different cytokines (INF-γ, IL-2-Th1; IL-4, IL-5,IL-13- Th2; IL-17,IL-22- Th17) which have proinflammatory and immunoprotective effects. CD8^+^ cells on the other hand detect antigens presented on the MHC I and generate a cytolytic T cell (CTL) response against the tumour cells. T-cell receptors (TCR) are stimulated by the antigens and are crucial for T-cell proliferation and differentiation which is supported by co stimulatory molecules, such as CD28. Ligands for CD28, CD80 (B7-1), CD86 (B7-2), are expressed by the APCs and are upregulated when these cells encounter an antigen. Different negative regulators e. g Cytotoxic T lymphocyte antigen 4 (CTLA4) and Programmed cell death 1 (PD1) act as checkpoint molecules against the immune response so as to curb the hyperactivation and preserve self-tolerance. These checkpoint molecules have similar characteristics to costimulatory molecules and thus prevent the activation of effector T cells. Tregs (CD4^+^ CD25^+^) express the CTLA4 and thus have immunosuppressive effects. Other molecules e.g., CD3, a T cell co receptor, also play a major role in activating a naïve T cell. Upon sufficient downstream stimulation, activated T-cells generate survival cytokines such as IL-2, IL-4 and IL-7 which help them to grow and proliferate. Upon activation, T cells target the cancer so that: 1) restimulation-induced cell death occurs due to strong acute antigenic stimulation, 2) T-cell exhaustion occurs to an unresponsive state as stimulation is weak, 3) decrease of antigen specific T cell population occurs due to diminishing cytokine and antigen stimulation.88 A small number of cells also convert into “Memory T-cells” stimulated by IL-7 and IL-15 and continue to propagate in the immune system to generate a memorised response against future encounters with the same antigen which can be beneficial for the success of prophylactic cancer vaccines in the clinic.

Relatively higher numbers of cDCs cells are found in skin compared to muscle and thus the transdermal route provides an apt delivery route for different cancer vaccines. (Tawde et al., 2016) Of these migratory cDC1 (CD103^+^) are the most potent target with the ability to acquire the antigens, mature and then migrate from the periphery to the lymphoid site to interact with naïve T-cells to generate CTL effector cells. (Kushwah and Hu, 2011) The dermal Langerin^+^ CD103^+^ cDC1s present antigens via MHC I pathway generating elicited CTL levels. (Kushwah and Hu, 2011) A list of cancer vaccines targeting OC that have been completed phase I/II clinical trial with published results is summarised in Table 2. Note that none of these have to date involved non-viral delivery of nucleic acids.

**TABLE 2:** Table of completed clinical trials showing promising results for various cancer vaccines in OC. All of these vaccines have been used as a second line of treatment after initial cytoreductive debulking of the primary tumours and chemotherapy.

Clinical trial ID	Cancer type	Vaccine description	Vaccine type	Therapeutic cargo	Additional intervention	Vaccine delivery route	No. of partici-pants	Phase	Remarks
NCT 01867086	Stage III/IV OC—Recurrent	Bi-shRNA-furin and GMCSF augmented autologous tumour cell Vaccine	Autologous whole tumour cell vaccine	RNA interference moiety -Bi-shRNA-furin	Drug: Carboplatinum Drug: Carboplatinum and Taxol	ID once every 3 weeks- Max 12 doses or as long as it lasts	1	II	All patients were ELISPOT-positive after 12 months (100%). This subject did not complete treatment due to disease progression. After 24 months, subject was not alive. Statistical analysis was not done. This study was terminated
NCT 01551745	Stage III/IV OC- Recurrent/Refractory	Bi-shRNA-furin and GMCSF augmented autologous tumour cell Vaccine	Autologous whole tumour cell vaccine	RNA interference moiety -Bi-shRNA-furin	Drug: Bevacizumab	ID once every 4 weeks -Max 12 doses or as long as it lasts	5	II	All patients were ELISPOT positive after 12 months (100%). 1 (20%) patient was alive after 2 years 3/5 showed some serious adverse events like hepatobiliary disorder, infections and infestations and nervous system disorder
NCT 01617629	EOC-stage III/IV	Cvac (MUC 1 Autologous dendritic cells pulsed with recombinant human fusion protein coupled to oxidized Polymannose)	Autologous Dendritic Cell Vaccine (MoDC)	Dendritic cells specific to MUC-1, Manosylated fusion protein		ID injections- every 4 weeks (dose1-3), every 12 weeks (3–6)	9	II	Due to the few patients, Overall Survival could not be calculated. All-cause mortality was observed 2/9 patients. Severe adverse events were seen in 22.22% patients with 77.78% patients having other adverse events
NCT 01068509	EOC- stage III/IV 1st or 2nd remission	Cvac (MUC 1 Autologous dendritic cells pulsed with recombinant human fusion protein coupled to oxidized Polymannose)	Autologous Dendritic Cell Vaccine (MoDC)	Dendritic cells specific to MUC-1, Manosylated fusion protein		ID injections- every 4 weeks for 24 weeks	63	IIb	Therapy was safe with only 7 patients. CVac-treated patients had T cells that responded to mucin 1 challenge seen with both CD4^+^ (helper T cells) and CD8^+^ (killer T cells). CD8^+^ cytotoxic T cells showed a greater reactivity than CD4^+^ T helper cells. Detectable mucin 1-specific T cell response in treated patients as compared to untreated that was measurable over endogenous baseline (unstimulated) suggested a prolonged immune response. Patients with 2nd clinical remission had longer PFS (13 vs. 5 months) and overall survival (>42 vs. 26 months) when compared with unvaccinated patients. 15.38% of the vaccinated patients suffered from serious adverse effects whereas other mild adverse events were observed in 96.15 % of the vaccinated patients
NCT 00091273	Stage I-IV EOC, Primary Peritoneal Cavity Cancer	Adjuvant vaccine comprising ovarian cancer synthetic peptides, tetanus toxoid helper peptide, and sargramostim (GM-CSF) emulsified in Montanide ISA-51	Peptide vaccine	Ovarian cancer synthetic peptide		SC and ID in 2 different sites (Day 1, 8, 15, 29, 36 and 43)	9	I	Measure of Tumor-antigen-specific Immunity in PBMC by ELISPOT Assay showed response in 8 patients even at 3 months. No serious adverse events or mortality were observed until day 50, however other mild adverse events were observed in all of the patients
NCT 00857545	Stage I-IV Fallopian Tube Cancer, Stage I-IV OC, Stage III/IV Primary peritoneal cancer In their 2nd/3rd remission	Polyvalent vaccine (including GM2-keyhole limpet hemocyanin [KLH], Globo-H-KLH, Tn-mucin 1 [MUC1]-32mer-KLH, and Thompson Friedreich antigen [TF]-KLH with Saponin-based immunoadjuvant OBI-821	Conjugate Peptide vaccine	Polyvalent carbohydrate/peptide antigens		SC once in weeks 1, 2, 3, 7, 11, 23, 35, 47, 59, 71, and 83	171	II	<50% of patients were found to have IgM + response to the individual antigens. IgG + responses ranged 7%–45%. MUC1 was the most immunogenic antigen, with 49% and 45% of patients developing a IgM and IgG response, respectively, when comparing the pre- and post-titers. 77% discontinued due to progression, 4% due to toxicity, and 1 due to myeloid dysplastic syndrome (MDS). Lesser adverse events were injection site reactions (82%) and fever (11%). Vaccination with this polyvalent construct with antibody effectors was modestly immunogenic but did not prolong PFS or OS when compared to OPT-821 alone
NCT 00616941	EOC, Fallopian Tube Cancer, Primary Peritoneal Cancer	Synthetic peptide vaccine encoded by NY-ESO-1 gene in combination with Montanide and polyinosinic-polycytidylic acid - poly-l-lysine carboxymethylcellulose (poly-ICLC)	Peptide vaccine	NY-ESO-1 encoding overlapping peptides- synthetic		SC injection once every 3 weeks for 5 doses	28	I	Vaccination induced an integrated immune response (CD4^+^-100% and CD8^+^ T 25%–90.9%) post baseline. OLP immunisation alone failed to induce CD4^+^ T-cell responses; instead, it reduced high-avidity CD4^+^ T-cell progenitors that had previously identified naturally processed NY-ESO-1 protein. High-avidity NY-ESO-1-specific CD4^+^ T-cell precursor growth needed OLP emulsification in Montanide. While inhibiting the generation of IL-4 producing Th2 and IL-9 producing Th9 cells, poly-ICLC greatly improved CD4^+^ Th1 responses
NCT 01223235	Fallopian Tubes Cancer, OC, Peritoneal Cancer	Polyvalent vaccine-KLH conjugate + OPT-821	Conjugate Peptide vaccine	Polyvalent carbohydrate/peptide antigens	Drug: Bevacizumab	SC once every week (Doses 1–3), once every 4 weeks (doses 4–6)	22		Bevacizumab improved the vaccine’s tolerability. Response was not linked to a higher chance of survival. Increased IL-8 was linked to a considerable improvement in PFS on a two-timepoint analysis. Cytokine levels were not substantially correlated with survival across all timepoint measures. 1 patient experienced toxicity that was dose-limited (grade 4 fever). 2 (10%) patients developed grade 3 hypertension as a result of bevacizumab. 13 (68%) and 16 (84%) of the 19 participants reacted to 3 and 2 antigens, respectively (Globo-H, GM2, TF cluster Tn, MUC-1). Out of the 21 patients, 4 were still living after more than 5 years
NCT 00112957	Fallopian Tube Cancer, OC, Peritoneal Cavity Cancer	Recombinant vaccinia-NY-ESO-1 (rV-NY-ESO-1) and recombinant fowlpox-NY-ESO-1 (rF-NY-ESO-1)	Recombinant viral nucleic acid vaccine	NY-ESO-1 encoding viral constructs		ID (rV-NY-ESO-1- day 1), SC injections of (rF-NY-ESO-1-once every 4 weeks- 6 doses	23	II	38% of patients were in remission at 1 year. Specific antibody response to the NY-ESO-1 and LAGE-1 measured by ELISA showed increase in response at different timepoints until 12 months when compared to Day 0. Detectable T-cell responses was observed in 90.9% of patients for CD4^+^ and 45.5% for CD8^+^ cells. Higher number of patients with release of INF-γ by T Cells (CD4^+^-75%, CD8^+^-25%) in response to cancer antigens was observed after vaccination at different timepoints until 12 months 2 patients had to be discontinued because of treatment emergent adverse events with 4 patients facing serious adverse events
NCT 00803569	Fallopian Tube Cancer, OC, Peritoneal Cavity Cancer	ALVAC(2)-NY-ESO-1(M)/TRICOM vaccine administered with the granulocyte macrophage-colony stimulating factor (GM-CSF) Sargramostim	Recombinant viral vector nucleic acid vaccine	Recombinant genes encoding NYESO-1(M), TRICOM (LFA-3, ICAM-1, B7.1), vvE3L, vvK3L		SC injection once every 4 weeks for 6 doses	13	I	The vaccine was well tolerated by all patients with no patients with discontinuation, mortality or any serious treatment related adverse effects. 83.3 % of the patients had no evidence of disease after 24 weeks, 16.7% patients were with progressive disease. Median PFS was observed to be 167.5 days 83.3% and 25% patients were found to have NY-ESO-1 and LAGE-1 antigen positivity post baseline through 24 weeks
NCT 00088413	Adenocarcinoma, Colorectal Cancer, OC, Breast Cancer	PANVAC-V (Vaccinia) and PANVAC-F (Fowlpox) containing the transgenes for CEA and MUC-1 in Combination With GMCSF	Recombinant viral vector nucleic acid vaccine	Recombinant genes encoding CEA and MUC 1		SC PANVAC-V-day 1 PANVAC(TM)-F once after every 2 weeks (Dose 2–4) then once every 4 weeks up to 12 doses	51	I/II	Side effects were largely limited to mild injection-site reactions. Ovarian cancer: For patients (*n* = 14), the median time to progression was 2 months (range: 1–6), and the median overall survival was 15.0 months ELISPOT assay of 2 HLA-A2+ ovarian cancer patients who were enrolled in the study for 2 months showed no significant changes without *in vitro* stimulation, for CEA nor MUC-1. However, after *in vitro* stimulation with HLA-A2 restricted CEA and MUC-1 peptides for 72 h, 1 of the 2 patients had a 2.7-fold increase in CEA-specific T-cells. Increases in the T _effector_: T_reg_ ratios were observed in 3 patients
NCT 02179515	Lung Cancer, Breast Cancer, Prostate Cancer, Tumours (Others), Ovarian Cancer	Modified vaccinia Ankara (MVA)-brachyury-B7-1, ICAM-1 (Intercellular Adhesion Molecule 1), and LFA-3 (lymphocyte function-associated antigen 3) TRICOM vaccine * *	Recombinant viral vector nucleic acid vaccine	Recombinant gene encoding Brachyury		SC injection once every month for max 6 months	38	I	Vaccination with 1 dose was not successful in generating any T-cell response after 85 days, however 35.7% and 60.0% patients developed a response after 2nd and 3rd dose respectively. Single dose did not generate any Anti-Brachyury Antibodies in any patient whereas a 2nd and a 3rd dose generated response in 7.1% and 26.1% patients respectively. Increased production of INF-γ was observed with 2 and 3 doses of the vaccine. Most of the patients suffered from post treatment adverse effects with 2/3, 4/17 and 2/18 patients having serious adverse effects after 1,2 and 3 doses
NCT 00623831	Melanoma, Sarcoma, Gastrointestinal Stromal Tumor (GIST), Head and Neck Cancer, Transitional Cell Carcinoma, Prostate Cancer, OC, Esophageal Cancer, Breast Cancer	Mixed bacterial vaccine (MBV, Coley’s toxin)	Mixed bacterial vaccine	Heat-inactivated *Streptococcus pyogenes* and *Serratia marcescens* Lysate		SC injection twice weekly for 6 weeks	17	I	13 patients were in cohort 1 (dose level 1) and 4 in cohort 2 (dose level 6). After receiving MBV, 11 of them experienced fever (cohort 1). The serum IL-6 levels increased consistently in 10 out of 12 patients, with the maximum levels occurring at the same time as the highest body temperatures. A subgroup of patients displayed rising TNF-, IFN-, and IL1- levels. The partial tumour response in a patient with metastatic bladder cancer was closely linked with MBV-induced fever and raised levels of numerous cytokines

### 4.2 Dendritic cell vaccines

DC based vaccines use autologous DC cells matured with whole tumour lysate containing the TAA to generate an anti-tumour T-cell response. The most common approach among the clinically registered trials for *ex vivo* differentiated DC vaccines relies on the methods of extraction and isolation of CD14^+^ monocytes (MoDCs) by the process of leukapheresis. (Kandalaft et al., 2013; Calmeiro et al., 2020) Immature MoDCs extracted from patients are matured *in vitro* in presence of IL-4 and GMCSF and exposed to patient specific TAA protein extracted after cytoreductive debulking, which results in the upregulation of the surface markers such as CD80, CD86, CD40 and also lymph node migratory receptors CCR7. These matured antigen presenting DCs are then reinjected into the patients to prime naïve T-cells. (Kandalaft et al., 2013) At present, over 300 clinical trials for DC based anticancer vaccines have been completed or are ongoing for different cancers among which ∼18 are specifically for OC. Based on the positive results in the previous Phase I and IIa trials Gray et al. (2016) conducted a Phase IIb clinical trial of Cvac, (DC-Manan-fusion protein) a MUC-1 autologous dendritic cell vaccine therapy, in multinational patients with confirmed stage III/IV OC. Immunoassay to assess T-cell response suggested a higher CD8^+^ CTL response than CD4^+^ T helper cells in patients receiving complete 10 doses of 6 × 10 (Crum et al., 2013) DCs/ml over 56 weeks. Higher MUC-1 specific T-cell responses were detected when compared to basal levels of patients receiving standard of care. They also found improved progression free survival; 13 months compared to 9 months. In Cvac treated patients and overall survival in patients in their 2nd clinical remission (13 months vs. 5 months) was higher compared to the ones in 1st remission (18 months vs. 13 months). (Gray et al., 2016) Even though these DC based vaccines haven been able to show some positive results, their success rates are usually lower than 15%. (Calmeiro et al., 2020) DC-based vaccines are often used as a secondary treatment to prolong the remission after a primary treatment of cytoreductive surgery and chemotherapy. They are based on the patient specific antigens that need to be identified from the debulked tumour to manufacture peptides or nucleic acids to pulse the DC *invitro*. The limitation associated with using this is that it might extend the time between the first line of treatment and the first dose up to several months. (Gray et al., 2016) They are also a very expensive (_∼_ £4,000 per single vaccine in European countries), labour intensive process specific to individual patients and the yield of autologous DCs from cancer patients for use in a vaccine is often problematic as during the harvesting process of MoDCs there might be contaminants such as Red blood cells (RBCs) and platelets along with it and also all the DC harvested might not mature during their exposure to the antigen at similar rates. In addition, the procedure requires a high-level clean room with GMP facilities for DC generation, *in vitro* maturation of the DCs may pose other difficulties such as risk of contamination, improper maturation, decreased cell longevity, inability of the DCs to reach the lymph nodes to elicit proper immune response, etc., (Turnis and Rooney, 2010) Thus, other vaccination methods to induce the naturally circulating DC in the patients could be preferred.

### 4.3 Peptide vaccines

One of the ways of exploring the immunogenic effects of naturally circulating DCs are peptide vaccines. These vaccines consist of immunogenic short peptide segments (usually of 20–30 amino acid sequence) of whole tumour antigens, which have the potential to initiate an immune response specific to the antigen. (Mittendorf et al., 2008; Brown et al., 2019) These antigenic peptides being exogenous in nature are taken up by the DCs (CD4^+^ cDC2) and presented on MHC II molecules which prime mostly a humoral response by the CD4^+^ T-cells. However, a small fraction of these peptides is also cross presented on the MHC I molecules by the CD4^+^ DCs to induce direct CTL response. These vaccines have been extensively investigated in different cancers. (Mittendorf et al., 2014; de Paula Peres et al., 2015; Kalli et al., 2018; Brown et al., 2019) Some peptides extensively studied in OC include human epidermal growth factor receptor 2 (HER2/neu), CEA and MUC-1 that have been able to show some positive immunogenic results. (de Paula Peres et al., 2015)

A Phase I/IIa clinical study by Brown et al. (2019) suggested reduction in the recurrence risk of ovarian and endometroid cancers when peptide vaccines (E39 plus GM-CSF) specific to folate binding proteins were administered intradermally to patients. A robust, dose-dependent *in vivo* immune response was seen in vaccinated patients in comparison to the control group. Disease free survival (DFS) was improved in vaccinated patients (55.5%) when compared to the control group (40.0%) after 24 months. The DFS was further seen to improve to 90.0% in patients receiving higher doses of 1,000 µg after receiving treatments of their primary disease but not in recurrent patients. Patients were also given boosters after 6 months to improve the DFS. Thus, the vaccine proved to be safe and was effective to certain extent to prevent recurrence of the disease in high-risk ovarian and endometroid cancer. Another potential target identified by Kalli et al. (2018) for a peptide vaccine that could be used for breast and ovarian cancers is folate receptor alpha (FRα). The peptides chosen were successful in generating a durable T-cell immunity specific to FRα in more than 90% patients (Breast cancer stages II, III and OC stages II- IV) in the phase I clinical trial of the vaccine. Modifications of the peptide with immunoadjuvants have been successful in generating high numbers of long lived CD8 memory T cells. A phase 1 clinical trial on ovarian cancer patients with NY-ESO-1 overlapping peptides (OLP4) revealed that when the peptide was used with adjuvants such as Montanide only and Montanide + polyinosinic-polycytidylic acid (PolyICLC) it elicited a better immune response than when it was used alone. Increased detectable IgG antibody levels after 4 months were seen in almost 91% of the patients receiving the vaccine with both adjuvants when compared 46% with one and 25% with peptide alone. It also showed that the peptide alone generated a CD4^+^ response in 100% of the cohort while the CD8^+^ response was seen only in 25% of the patients. However, this increased to 62% and 91% with the adjuvants without effecting the CD4^+^ response. (Sabbatini et al., 2012) Peptide vaccines have the advantages of relative ease of preparation economically without having the patients go through additional procedures as in the case of DC cell-based vaccines. However, short oligopeptide vaccines might exhibit antigen induced cell death of effector T-cells due to overstimulation by the same immunogenic peptide. (Berry et al., 2017) Also the oligo peptides bind to the MHC I complexes of other somatic cells other than APCs which stimulates suboptimal T-cell priming. Full length protein vaccines however can be processed and presented only by the APC in form of antigenic peptides stimulating optimal T-cell response, but these proteins pose the hindrance of being efficiently chemically produced, endocytosed, and processed inside the APC. Both long and short peptides have limited ability to overcome the biological barriers and are highly sensitive and degradable when exposed to different body fluids and enzymes. (Myc et al., 2011) Another reason for the limited success of peptide vaccines is the high response generation of short lived humoral immunity compared to cellular response because of poor uptake by the cDC1 APC. Also, the naïve T-cell tend to differentiate more into Th2 cells in presence of IL-4 and IL-2 instead of Th1 cells which help in inducing more antibody mediated immunity rather than cell mediated immunity. (Speiser et al., 2002) Thus, nucleic acid vaccines can provide an alternative to generate more cellular cytolytic response against tumour cells.

### 4.4 Nucleic acid vaccines

Nucleic acid vaccines aim to deliver genetic material that encodes tumour antigens into the host, where they are taken up by innate immune cells and are transcribed and translated (DNA) or translated (mRNA) to produce the antigenic protein. This ultimately leads to the presentation of the tumour antigenic peptide fragments to adaptive immune cells. DNA vaccines use engineered plasmid DNA (pDNA) encoding the antigen that is delivered into the patient, normally via intradermal/subcutaneous or intramuscular injection. However, Intradermal routes have been proven to elicit strong Th1 and CD8^+^ cytolytic T-cell response along with humoral response when compared to other delivery routes. (Rahman et al., 2000; Constantino et al., 2016) The success rate of these vaccines is highly dependent on the ability of the therapeutic nucleic acid material to overcome the cellular environment to reach the nucleus of the target cell. Cellular barriers include the plasma membrane, endosomal membrane, and the nuclear membrane. Anionic lipophilic cell membranes restrict the entry of the negatively charged hydrophilic genetic cargo such as plasmid DNA. Although endocytosis might aid cellular entry, endosomal entrapment becomes an issue. Nuclear membranes also pose a barrier for pDNA cargos which need to undergo transcription in the nucleus and then transport back to cytosol for translation (Wang et al., 2012).

**FIGURE 4:**
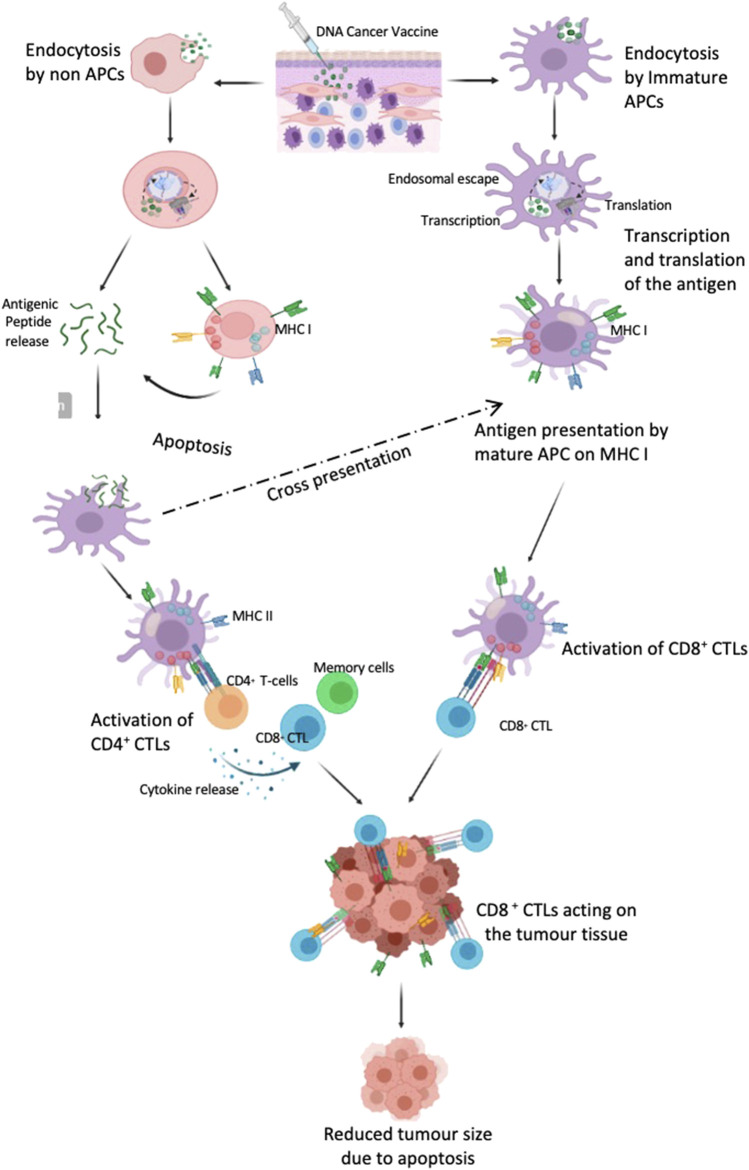
DNA vaccine targeting DC cells to induce an immune response. The vaccine is delivered intradermally where the immature APC, macrophages and other immune cells are recruited. The cargo enters the immature APC, traffics to the nucleus where the RNA transcription occurs followed by protein translation in the cytoplasm and is then represented on the cellular surface as MHC I and thus matures the DC. These cells then trigger the CD8^+^CTL response. However, if the plasmid is taken up by the non-APC cells, they either present them in MHC I and subsequently release the antigenic peptide on apoptosis or directly release the peptide by proteolysis by proteosome. These peptides are then taken up by the APC and either cross presented in MHC I or presented on MHC II which stimulates the CD4^+^ T cell response which further generates CD8^+^ CTL and memory cells. (Created with BioRender.com).

Once the pDNA enters the cells it may be exposed to one of the following fates (Figure 4): 1) it enters into a APC (preferably cDCs) transported to the nucleus of DCs to initiate the mRNA transcription followed by the synthesis of antigenic protein in the ribosome, degraded in the proteosome and ultimately be presented on the cell surface from the endoplasmic reticulum/golgi bodies in an MHC I (8–10 amino acid lengths) to stimulate antigen specific CD8^+^ CTL 2) pDNA is taken up by non APCs which then process it and either present it in MHC I molecules subjecting it to apoptosis later and release of the peptides or releases the peptides directly by lysosomal degradation. The released peptides are then either taken up by the DC or B lymphocytes to present it in MHC II (13–25 amino acid length) and later prime CD4^+^ T helper cell response or generate antibodies for a humoral response. RNA vaccines work in similar pattern except for that they do not require transcription and only require delivery to the cytoplasm to be translated.

Unlike peptide vaccines, DNA vaccines are competent in generating both a humoral and cellular immune response *in vivo*. Alfredo, et al. were able to generate a robust CD4^+^ and CD8^+^ cellular immune response against ovarian cancer in murine models with a DNA vaccine targeting follicle-stimulating hormone receptor (FSHR) in an pVAX1 expression vector with three doses administered 2 weeks apart using electroporation. Higher expression of INF-γ, IL-2 and TNF-α was observed after vaccination when compared to empty vector controls. The vaccine was also successful in generating a strong humoral antibody response against the FSHR transmembrane protein and a long-lasting cellular response with CD4^+^ and CD8^+^ cells producing INF-γ, IL-2 and TNF-α at equivalent levels even after 3 months of complete immunisation as in the first week after the last vaccination. The study suggested employment of this vaccine as a second line of treatment to prevent recurrence after debulking of the ovaries as primary standard care. (Perales-Puchalt et al., 2019) A randomised phased II trial of PANVAC, a viral DNA vaccine consisting of human genes expressing CEA and MUC-1 and 3 T-cell costimulatory molecules in metastatic breast and ovarian cancer, showed improved progression free survival in patients with metastatic breast cancer and ovarian cancer when used in combination with other chemotherapy. (Heery et al., 2015) The patients with limited disease burden and a less compromised immune system seem to benefit from the vaccine with increased ratio of T_effectors_: T_regs_. (Mohebtash et al., 2011; Heery et al., 2015) Even though various clinical trials have been ongoing, no nucleic acid based vaccine has been approved for treating ovarian cancer in human as of yet.

Nucleic acid-based vaccines are economic, easy to produce and purify compared to peptide antigenic vaccines. They are easily re-designable, safe to administer and also highly stable (Restifo et al., 2000) with DNA vaccines being more stable than the RNA vaccines. Cold storage is necessary in most of the vaccine strategies to guarantee the stability and longevity of the vaccines. As shown with the recent mRNA vaccines e.g., mRNA-1273 (Moderna Inc. Cambridge, MA, United States) for COVID-19 a cold storage temperature of −20°C is necessary. However, DNA vaccines are more highly durable and require minimal refrigeration temperatures, making them extremely useful. Significant stability for over 24 months was observed with lyophilised pDNA stored at higher temperatures at + 2°C–8°C. (Van der Heijden et al., 2013) Unlike peptide vaccines, DNA vaccines are not Human leukocyte antigen restricted (HLA) and can be presented on the MHC I and MHC II robustly. However, naked nucleic acid vaccines are inefficient to generate the required immunological response as they do not pass through the plasma membrane and into the cells freely. Thus, different gene delivery mechanisms are used to generate an adjuvant effect and enhance the functionality of the DNA vaccines.

### 4.5 Nucleic acid vaccine delivery

To overcome the biological barriers different methods have been investigated by researchers to assist the transfer of nucleic acid molecules. Physical methods such as electroporation, where pulsed electric fields are applied to increase the permeabilization of the cellular membrane in order to increase the uptake of the therapeutic cargo have proven to be successful. However, intense high voltage pulses may cause irreversible damage to the cells and other important organs *in vivo*. (Sokołowska and Błachnio-Zabielska, 2019) Other physical methods such as gene gun, (Kandalaft et al., 2013) sonication by ultrasound (Rahman et al., 2000; Wang et al., 2012) etc., have also been used to deliver nucleic acids (DNA) in to cells but have not been fully successful in generating a strong immune response due to lower transfection efficiency *in vivo* and rapid degradation of DNA by sonication. Thus, use of different vectors to deliver the gene are taken into consideration.

The vectors used for the delivery of the therapeutic nucleic acid must have the ability to condense the nucleic acid cargo protecting it from the degradation, opsonisation and expulsion from the systemic circulation to stimulate cellular internalisation by avoiding endosomal entrapment and promoting nuclear import to the targeted cells. Viral vectors are one of the most widely used delivery mechanisms for gene delivery that have undergone clinical trials for Ovarian cancer (Completed trials with results-NCT00112957, NCT00803569, NCT00088413, NCT02179515- Table 2). However, limitations including toxicity, restriction in nucleic acid loading, immunogenicity and difficulties in mass production have led researchers to search for new alternative non-viral strategies. Table 3 lists the different strategies used to deliver nucleic acid cargo to cells.

**TABLE 3:** Table of different strategies used to deliver nucleic acid cargo to cells.

Sl.no.	Type of delivery	PROS	CONS
1	Physical Strategies	No restrictions on the length of the coding sequence that can be carried by the physical vectors, no side effects associated with viral or biochemical methods, and direct penetration of both small and large nucleic acid molecules into the cytosol	May cause cell rupture, nucleic acid degradation, requires extra equipment, generates lower immunogenic response
2	Viral Delivery	Long-term gene expression via prolonged replication (e.g., adeno-associated virus) or gene integration into the host genome through established mechanisms for cellular absorption, Higher immunological response, nucleic acid release and nuclear transport, and (e.g., retroviruses)	May cause adverse immunogenic reactions, chances of recombination and mutagenesis, may be cytotoxic, needs cautious preparation, limitation in repeated administrations, delayed immune response
3	Non-Viral strategies	Simple preparation, negligible immunogenicity and oncogenicity, and no probability of recombination	Level of uptake might be lower, gene expression is short lived, may cause adverse effects due residual substances

#### 4.5.1 Adjuvants for nucleic acids

Adjuvant literally is derived from the word “adjuvare” meaning help in Latin. Thus, an adjuvant may be defined as any substance that aids in the function of the vaccine to enhance its immune response against any antigen by recognising the damage associated molecular patterns (DAMPs) and the pathogen associated molecular patterns (PAMPs) by the pattern recognition receptors (PRRs) of the innate immune cells (Akira et al., 2006; Kawai and Akira, 2010; Takeuchi and Akira, 2010) which surges the antigen specific immune response inducing powerful innate and adaptive responses against any tumour cells or any other pathogen entering the body. Another possible way in which an adjuvant functions may also be as a delivery mechanism to effectively deliver the antigen to the antigen presenting cells (APCs) to increase both the innate and adaptive immune response specific to the antigen.

Advancement in immunological research have been able to establish that the various vaccine adjuvants can act by one or more of the following mechanism to evoke a immune response: 1) it may cause a depot effect to cause a sustained release of the cargo at the injection site; 2) recruitment of immunogenic cells at the injection site; 3) increase the uptake of the cargo and its presentation to the APC; 4) activation and maturation of the APC with expression of MHC class II and other co stimulatory molecules and migration to the lymph nodes; 5) chemokines/cytokine upregulation. (Cox and Coulter, 1997; Hoebe et al., 2004) Some of the examples of nonviral adjuvants include nanoparticles such as gold particles, lipid based vesicles, mineral oil based emulsions, and water based emulsions, alum, empty bacterial cell envelopes etc., (Edelman, 2002; Petrovsky, 2006; Muhammad et al., 2012) Very few of these adjuvants have been used to deliver nucleic acid cargos for OC treatment (Table 4). However, while selecting a suitable adjuvant for a vaccine strategy the following has to be taken into consideration 1) route of delivery of the vaccine 2) immune status of the host 3) timing and dose of the vaccine 4) construction of the antigen to be delivered 5) formulation of the adjuvant, 6) size of the adjuvants.

**TABLE 4:** Table of clinical trials showing adjuvants used to accelerate Nucleic acid delivery for various cancer vaccines in OC.

Clinical trial number	Status	Type of treatment	Adjuvant used to accelerate nucleic acid delivery	Efficacy of vaccine
NCT00112957	Completed	Therapeutic (2nd line)	Recombinant Vaccinia and Fowlpox viruses expressing NY-ESO-1 or LAGE-1	38%
NCT00803569	Completed	Therapeutic (2nd line)	Recombinant canarypox virus encoding NY-ESO-1 and Co-stimulatory Molecules (B7-1, ICAM-1 and LFA-3)	83%
NCT00088413	Completed	Therapeutic (2nd line)	Primed with recombinant Vaccinia and boosted with Fowlpox viruses to deliver CEA and MUC-1 with GM-CSF	All 14 patients in OC group had progressive disease and did not complete the study
NCT02179515	Completed	Therapeutic (2nd line)	Modified Vaccinia Ankara Virus expressing the TRICOM of B7-1, ICAM-1 and LFA-3	1 dose-0%
				2 dose-36%
				3 dose-60%
NCT04163094	Active, not recruiting	Therapeutic (Before and long with 1st line of chemotherapy)	Liposomal delivery of three OC TAAs in combination with Neo-adjuvant chemotherapy	No results posted yet
NCT02275039	Completed	Therapeutic (2nd line)	Modified Vaccinia Ankara Virus expressing P53 with Gemcitabine	No results posted yet
NCT00436254	Active, not recruiting	Therapeutic (2nd line)	Plasmid delivered with GM-CSF	No results posted yet

#### 4.5.2 Cell penetrating peptides as adjuvants

Cell penetrating peptides (CPP) (Cole et al., 2017; Feni and Neundorf, 2017; Habault and Poyet, 2019) are a lesser known non-viral strategy that can be employed in vaccines to deliver nucleic acids to the target cells. (Lee and Foote, 2009) This alternative mechanism include advantages of easy preparation, low immunogenic and oncogenic characteristics with no potential chance for recombination. They can be considered as a potential adjuvant for a vaccine provided they successfully condense the anionic nucleic acid cargo into a nanoparticle and deliver it successfully into APCs.

Targeting uptake of vaccine cargo into DCs and other cells is heavily dependent on the shape, size and charge of the cargo delivered. The use of nanoparticles is supported by the presumption that a higher cellular uptake of the antigenic cargo and an enhanced interaction with the immune cells can be achieved by an ideal optimised delivery system. (Neek et al., 2019) Degradation of the nucleic acid cargo by internal enzymes such as DNAase and RNAase can be averted by the application of biocompatible nanoparticles which encapsulate the cargo. The particles below 0.5 µm are easily taken up by the DCs (Foged et al., 2005) which then travel to the lymph nodes and induce an adaptive immune response. CPP NPs are self-assembled and have a similar shape and size similar to viral particles with the potential to induce the suitable immune responses. CPPs are small amino acid sequences (5–30 amino acids) with the ability to penetrate through the extra and intracellular barrier of the cells to release the nucleic acid cargo at the destination site. (Feni and Neundorf, 2017; Habault and Poyet, 2019).

The CPP nanocomplexes are easily manufactured to induce the desirable characteristics to the NP by changing their basic amino acid backbone. After the first discovery in 1988, many CPPs have been designed so far in the recent years to act as delivery system for proteins, therapeutic nucleic acid and other small organic molecules. They were often fused with different antigens or are used in DNA vaccines to facilitate the transport of the cargo, increase APC uptake and presentation. CPPs such as TAT, hPP70 have been successful previously in delivering nucleic acid cargos to specific cells for the treatment of various cancers through immunotherapy. (Niu et al., 2018; Ding et al., 2019) Previously, CPPs have also been used as DNA vaccine strategies to deliver DNA cargo to cells. A DC targeting vaccine strategy with microneedles loaded with CPP-PEI_1800_-Man/DNA complexes encoding Trp_2_, GMCSF and Fc genes was successful in inducing an elevated GFP^+^ CD11c^+^ DC response in lymph nodes (42.2%) and spleen cells (49.6%) of vaccinated BALB/c mice when compared to controls. The mice vaccinated with three doses of the vaccine at weekly intervals were subjected to tumour challenge after 1 week of final immunisation which resulted in 90% survival of all mice during the period with 68.5 % inhibition rate of tumour growth with a reduced tumour volume of 297.2 mm^3^ (control group tumour volume = 943.8 mm (Ahmed et al., 2020)) after 35 days of the challenge. The INF-γ concentration was also increased almost 4 -fold and a 9-fold increase in levels of IL-2 in vaccinated mice compared to other controls. Therapeutic results of the vaccine with the CCP inhibited growth rate to 48% thereby reducing the tumour volume to almost half as compared to control group. (Mccarthy et al., 2014) Hung et al., used one such CPP VP22, an Herpes simplex Virus (HSV-1) protein to form spherical particles with 0.3–1 µm range to act as a peptide vaccine to deliver DNA encoding antigenic peptide HPV-16 E7 which is associated with most cervical cancers, to target cells. When C57BL/6 mice were vaccinated intradermally via gene gun, a 50-fold increase in the E-7 specific INF-γ^+^/CD8^+^ T cell precursors and a very high CTL response as compared to wild type DNA alone controls of E7 was seen. Vaccinated mice remained tumour free even after 63 days of tumour challenge whereas all other controls of unvaccinated mice and E7- DNA alone developed tumour after 14 days of the challenge. The therapeutic effects of the vaccine also exhibited lower number of pulmonary nodules compared to unvaccinated and wild type E-7 DNA controls when it was used to treat tumour metastases in the lungs. (Hung et al., 2022) All these studies thus predict that the use to CPP in vivo models can be successful in delivering a nucleic acid cargo. However, use of these CPP to deliver nucleic acid cargos in human clinical trials has yet to be explored.

#### 4.5.3 RALA as a nucleic acid carrier

**FIGURE 5:**
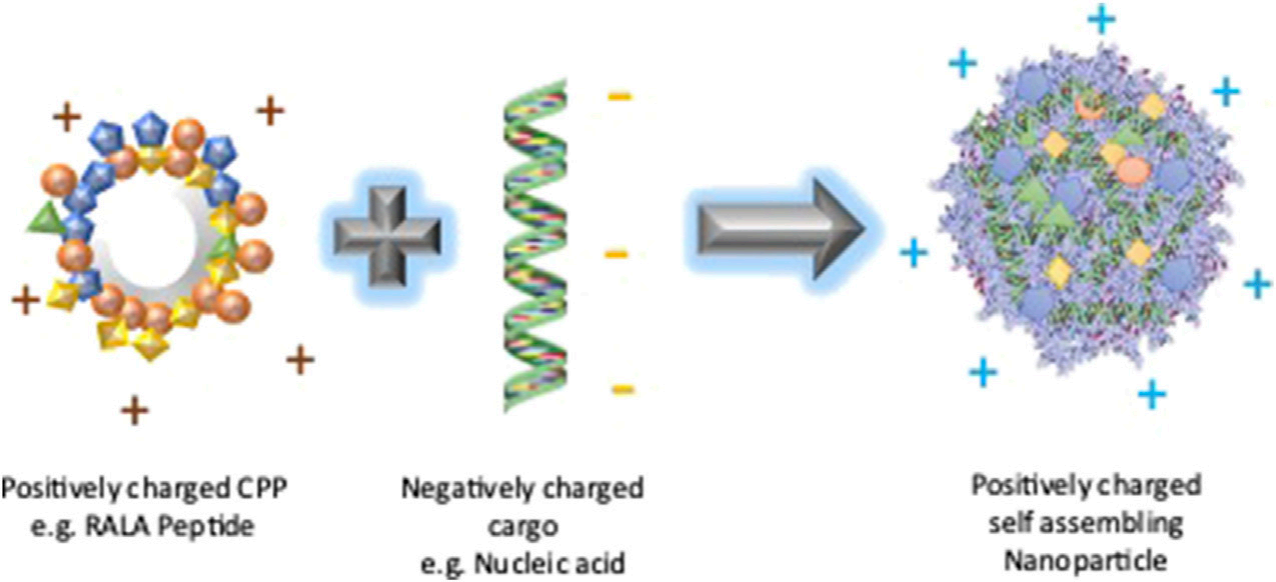
RALA Nanoparticle formation. The cationic RALA peptide forms a self-assembled nanoparticle with a negatively charged nucleic acid cargo due to the electrostatic interaction between them. The resultant nanoparticle is expected to have a positive charge due to the neutralization of the negative charge of the nucleic acid by the positively charged RALA.

RALA is one such synthetic cationic fusogenic CPP which can form self-assembling NPs with a negatively charged nucleic acid cargo (Figure 5). It is composed of 30 amino acid sequence:

N-WEARLARALARALARHLARALARALRACEA

The amino acids at specific sites have important effects on the CPP characteristics. Arginine sites (R), which are hydrophilic in nature, help to bind nucleic acids, leucine sites (L), which are hydrophobic in nature, bind with the lipid membranes, alanine (A) regions contribute to amphipathicity, tryptophan can be used as a spectroscopic probe and glutamic acid increases solubility in water at a physiological pH. (Mccarthy et al., 2014) The structure allows the peptide to bind with the lipid bilayer of the membrane of an endosome leading to an internal stress to cause pore formation and within the endosome when the pH drops around to 5.5 it adopts an alpha-helical structure by interacting with the phospholipid membrane enabling fusion with the endosome and effective release of the cargo to the cytoplasm. (Mcerlean et al., 2015).

RALA/pDNA nanoparticles with <100 nm size and a positive charge of _∼_+ 29 mV were produced successfully by Mccarthy et al. (2014) which were all within the specifications required for optimal cellular uptake of particles by endocytosis repressing the negative cellular membrane barrier. (Foged et al., 2005) RALA/mRNA complexes selectively disrupt membrane at acidic pH and successfully transfected cells *in vitro*. (Udhayakumar et al., 2017) No significant improvement in transfection efficiency when chloroquine—an endosomal disrupting agent is added is predictive that RALA is able to deliver the mRNA cargo (eGFP) on its own to the cytosol. (Udhayakumar et al., 2017) Characterisation of the particles suggested RALA to be a good nucleic acid carrier with higher α helicity, higher responsiveness to low pH present in the endosome, negligible toxicity effect *in vitro* and successful delivery of gene *in vivo* murine models. Negligible effects on circulating IgG, IgM, IL1-β and IL6 in mice was observed with RALA/pEGFP-N1 nanoparticles indicating that RALA itself does not provoke a neutralizing antibody response. (Mccrudden et al., 2018) Studies have also been able to establish functionality of lyophilised RALA nanoparticles which can be stored at room temperature. Cole et al. (2019) successfully delivered RALA/pDNA nanoparticles loaded in dissolvable PVA microneedles generating positive results against cervical and prostate cancer. (Lee and Foote, 2009; Van der Heijden et al., 2013; Cole et al., 2018b) Lyophilisation of RALA-pDNA (pE7) nanoparticles increased the size but kept within >100 nm with minimal change in the positive zeta potential, encapsulation efficiency (>80%), cell viability (80%). These fresh and lyophilised NP showed apparent protein expression when compared to pDNA alone indicating role of RALA to achieve transfection. These RALA NP, when incorporated in the polyvinyl alcohol (PVA) microneedles (MN), used to immunize mice once per 2 weeks (2–3 times), raised levels of anti -E7 IgG (80.06 µg/ml- two doses) compared to naïve controls (25 μg/ml). The splenic T cells from the MN immunized mice showed almost double cytolytic effects on tumour cells when compared to untreated control. RALA/pDNA NP when vaccinated through intramuscular injections and NP loaded in MN were able to reduce the established tumour growth in murine models indicating higher antigen specific cytolytic T-cell response when compared to pDNA on its own. (Cole et al., 2018b)

Udhayakumar et al. (2017) reported that RALA/mRNA OVA complexes were successful to produce elicited antigen specific CD8^+^ T cell response *in vivo* in murine models at N:P 5 and 10 when compared to mRNA alone thus indicating the role of RALA in generating a cytolytic cellular immune response. In the preliminary *invitro* studies mRNA complexed with RALA at N:P 10 was able to promote DC activation and upregulate CD86, CD40 and MHC II when compared to noncomplexed mRNA. DC activated with modified mRNA was less potent in comparison to the unmodified mRNA even though the levels of CD86, CD40 and MHC II were elevated compared to the PBS treated controls. RALA complexes at N:P 10 with modified mRNA reduced levels INF-β while leaving IL-6 levels unaffected with greater ability to elicit CD8^+^ T cell response and eliminate all target cells compared to unmodified mRNA. RALA/mRNA vaccine was compared with a conventional lipoplex (1,2-dioleoyl-3-trimethylammonium-propane (DOTAP)/1,2- dioleoyl-sn-glycero-3-phosphoethanolamine (DOPE))-based mRNA vaccine for their ability to elicit a CTL response *in vivo* intradermally in wild type (WT) mice and mice lacking the common INF-α/β receptor (Infar^−/−^). RALA/mRNA complexes (N:P 10) with unmodified mRNA showed higher responses for CTL after prime-boost immunization in Infar^−/−^ mice and similar levels with modified mRNA when compared with the WT mice indicating the negligible impact of Type I INF with modified mRNA/RALA complexes. Further investigation monitoring INF-β induction in INF-β reporter mice strain revealed a very high INF-β response in noncomplexed and DOTAP/DOPE complexed (N:P 1) modified mRNA explaining the elevated T cell immunity in Infar^−/−^ mice but when it was complexed with RALA (N:P 10) it hardly evoked any INF-β response and thus circumvents any negative effect of the type I INF without any additional augmentation of T-cell immunity in Infar^−/−^ upon vaccination. It was further proven that the non-immunogenic properties of RALA and its ability to induce substantial CD8^+^ CTL response *in vivo* is a unique feature which make it an effective delivery system for a vaccine strategy. (Udhayakumar et al., 2017) All the results thus obtained so far indicate RALA to be a strong candidate to act as a nucleic acid vaccine adjuvant.

## 5 Future perspective and conclusion

HGSC is the most aggressive type of OC, claiming the highest number of lives of the patients with OC, owing to its delayed diagnosis. Advances in sequencing techniques of the tumours are enabling effective identification of tumour antigens and some specific potential neoantigens as well. The genes encoding the overexpressed CTAs can be used as a potential target in a nucleic acid vaccine to induce a strong cytolytic CD8^+^ T-cell mediated immune response along with some humoral response in HGSC models *in vivo*. However, the inter and intracellular barrier of the cells pose a significant hindrance in delivering the negatively charged naked nucleic acid cargo to the cells thereby not probing the required immune response. CPPs can act as a potent delivery system required to successfully deliver the cargo and generate the immune response by transfecting the correct DC (cDC). There is no commercially available cancer vaccine to date for ovarian cancer. Nucleic acid vaccines provide platform for future clinical trials in HGSC; however, a non-immunogenic effective delivery system is required so that the immune response comes from the nucleic acid cargo itself.

## References

[B1] Agarwal S. Saini S. Parashar D. Verma A. Sinha A. Jagadish N. (2013). The novel cancer-testis antigen A-kinase anchor protein 4 (AKAP4) is a potential target for immunotherapy of ovarian serous carcinoma. Oncoimmunology 2 (5), e24270. 10.4161/onci.24270 23762804 PMC3667910

[B2] Ahmed N. Kadife E. Raza A. Short M. Jubinsky P. T. Kannourakis G. (2020). Ovarian cancer, cancer stem cells and current treatment strategies: A potential role of magmas in the current treatment methods. Cells 149 (3), 719. 10.3390/cells9030719 PMC7140629 32183385

[B3] Akira S. Uematsu S. Takeuchi O. (2006). Pathogen recognition and innate immunity. Cell 124, 783–801. 10.1016/j.cell.2006.02.015 16497588

[B4] Antoniou A. Pharoah P. D. Narod S. Risch H. A. Eyfjord J. E. Hopper J. L. (2003). Average risks of breast and ovarian cancer associated with BRCA1 or BRCA2 mutations detected in case series unselected for family history: A combined analysis of 22 studies. Am. J. Hum. Genet. 72 (5), 1117–1130. 10.1086/375033 12677558 PMC1180265

[B5] Barger C. J. Zhang W. Sharma A. Chee L. James S. R. Kufel C. N. (2018). Expression of the POTE gene family in human ovarian cancer. Sci. Rep. 8 (1), 17136. 10.1038/s41598-018-35567-1 30459449 PMC6244393

[B6] Batchu R. B. Gruzdyn O. V. Moreno-Bost A. M. Szmania S. Jayandharan G. Srivastava A. (2014). Efficient lysis of epithelial ovarian cancer cells by MAGE-A3-induced cytotoxic T lymphocytes using rAAV-6 capsid mutant vector. Vaccine 32 (8), 938–943. 10.1016/j.vaccine.2013.12.049 24406390

[B7] Beirne J. P. McArt D. G. Roddy A. McDermott C. Ferris J. Buckley N. E. (2019). Defining the molecular evolution of extrauterine high grade serous carcinoma. Gynecol. Oncol. 155 (2), 305–317. 10.1016/j.ygyno.2019.08.029 31493898

[B8] Bera T. K. Fleur A. S. Lee Y. Kydd A. Hahn Y. Popescu N. C. (2006). POTE paralogs are induced and differentially expressed in many cancers. Cancer Res. 66 (1), 52–56. 10.1158/0008-5472.can-05-3014 16397215

[B9] Berry J. S. Vreeland T. J. Hale D. F. Jackson D. O. Trappey A. F. Greene J. M. (2017). Evaluation of attenuated tumor antigens and the implications for peptide-based cancer vaccine development. J. Cancer. 8 (7), 1255–1262. 10.7150/jca.16450 28607601 PMC5463441

[B10] Braun M. W. Iwakuma T. (2016). Regulation of cytotoxic T-cell responses by p53 in cancer. Transl. Cancer Res. 5 (6), 692–697. 10.21037/tcr.2016.11.76 28944167 PMC5607642

[B11] Brown T. A. Byrd K. Vreeland T. J. Guy | Clifton T. Jackson D. O. (2019). Final analysis of a phase I/IIa trial of the folate-binding protein-derived E39 peptide vaccine to prevent recurrence in ovarian and endometrial cancer patients. Cancer Med. 8 (10), 4678–4687. 10.1002/cam4.2378 31274231 PMC6712444

[B12] Brunette L. L. Mhawech-Fauceglia P. Y. Ji L. Skeate J. G. Brand H. E. Lawrenson K. (2018). Validity and prognostic significance of sperm protein 17 as a tumor biomarker for epithelial ovarian cancer: A retrospective study. BMC Cancer 18 (1), 970. 10.1186/s12885-018-4880-x 30309325 PMC6182788

[B13] Calmeiro J. Carrascal M. A. Tavares A. R. Ferreira D. A. Gomes C. Falcão A. (2020). Dendritic cell vaccines for cancer immunotherapy: The role of human conventional type 1 dendritic cells. Pharmaceutics 12 (2), 158. 10.3390/pharmaceutics12020158 PMC7076373 32075343

[B14] Carter J. H. Deddens J. A. Mueller G. Lewis T. G. Dooley M. K. Robillard M. C. (2018). Transcription factors wt1 and p53 combined: A prognostic biomarker in ovarian cancer. Br. J. Cancer 119 (4), 462–470. 10.1038/s41416-018-0191-x 30057405 PMC6134086

[B15] Chen C. Liu J. Xu G. (2013). Overexpression of PIWI proteins in human stage III epithelial ovarian cancer with lymph node metastasis. Cbm 13 (5), 315–321. 10.3233/cbm-130360 PMC12928306 24440970

[B16] Chen Y. T. Hsu M. Lee P. Shin S. J. Mhawech-Fauceglia P. Odunsi K. (2009). Cancer/testis antigen CT45: Analysis of mRNA and protein expression in human cancer. Int. J. Cancer 124 (12), 2893–2898. 10.1002/ijc.24296 19296537

[B17] Cole G. Ali A. A. Mccrudden C. M. Mcbride J. W. Mcca J. Robson T. (2018). DNA vaccination for cervical cancer : Strategic optimisation of RALA mediated gene delivery from a biodegradable microneedle system. Eur. J. Pharm. Biopharm. 127, 288–297. 10.1016/j.ejpb.2018.02.029 29510205

[B18] Cole G. Ali A. A. McCrudden C. M. McBride J. W. McCaffrey J. Robson T. (2018). DNA vaccination for cervical cancer: Strategic optimisation of RALA mediated gene delivery from a biodegradable microneedle system. Eur. J. Pharm. Biopharm. 127, 288–297. 10.1016/j.ejpb.2018.02.029 29510205

[B19] Cole G. Ali A. A. Mcerlean E. Mulholland E. J. Short A. Mccrudden C. M. (2019). Acta Biomaterialia DNA vaccination via RALA nanoparticles in a microneedle delivery system induces a potent immune response against the endogenous prostate cancer stem cell antigen. Acta Biomater. 96, 480–490. 10.1016/j.actbio.2019.07.003 31299353

[B20] Cole G. McCaffrey J. Ali A. A. McBride J. W. McCrudden C. M. Vincente-Perez E. M. (2017). Dissolving microneedles for DNA vaccination: Improving functionality via polymer characterization and RALA complexation. Hum. Vaccines Immunother. 13 (1), 50–62. 10.1080/21645515.2016.1248008 PMC5287311 27846370

[B21] Coscia F. Lengyel E. Duraiswamy J. Ashcroft B. Bassani-Sternberg M. Wierer M. (2018). Multi-level proteomics identifies CT45 as a chemosensitivity mediator and immunotherapy target in ovarian cancer. Cell 175 (1), 159–170. e16. 10.1016/j.cell.2018.08.065 30241606 PMC6827878

[B22] Cox J. C. Coulter A. R. (1997). Adjuvants--a classification and review of their modes of action. Vaccine 15 (3), 248–256. 10.1016/s0264-410x(96)00183-1 9139482

[B23] Crum C. P. Herfs M. Ning G. Bijron J. G. Howitt B. E. Jimenez C. A. (2013). Through the glass darkly: Intraepithelial neoplasia, top‐down differentiation, and the road to ovarian cancer. J. Pathology 231, 402–412. 10.1002/path.4263 PMC3947463 24030860

[B33] Constantino J. Gomes C. Falcão A. Cruz M. T. Neves B. M. (2016). Antitumor dendritic cell-based vaccines: lessons from 20 years of clinical trials and future perspectives. Transl. Res.: J. lab. clin. med. 168, 74–95. 10.1016/j.trsl.2015.07.008 26297944

[B24] Daudi S. Eng K. H. Mhawech-Fauceglia P. Morrison C. Miliotto A. Beck A. (2014). Expression and immune responses to MAGE antigens predict survival in epithelial ovarian cancer. PLoS One 9 (8), e104099. 10.1371/journal.pone.0104099 25101620 PMC4125181

[B25] de Paula Peres L. da Luz F. A. C. dos Anjos Pultz B. Brígido P. C. de Araújo R. A. Goulart L. R. (2015). Peptide vaccines in breast cancer: The immunological basis for clinical response. Biotechnol. Adv. 33, 1868–1877. 10.1016/j.biotechadv.2015.10.013 26523780

[B26] de Visser K. E. Eichten A. Coussens L. M. (2006). Paradoxical roles of the immune system during cancer development. Nat. Rev. Cancer 6 (1), 24–37. 10.1038/nrc1782 16397525

[B27] Demir L. Yigit S. Sadullahoglu C. Akyol M. Cokmert S. Kucukzeybek Y. (2014). Hormone receptor, HER2/NEU and EGFR expression in ovarian carcinoma - is here a prognostic phenotype? Asian Pac. J. Cancer Prev. 15 (22), 9739–9745. 10.7314/apjcp.2014.15.22.9739 25520097

[B28] Deniger D. C. Pasetto A. Robbins P. F. Gartner J. J. Prickett T. D. Paria B. C. (2018). T-Cell responses to TP53 "hotspot" mutations and unique neoantigens expressed by human ovarian cancers. Clin. Cancer Res. 24 (22), 5562–5573. 10.1158/1078-0432.ccr-18-0573 29853601 PMC6239943

[B29] Ding Y. Zhao X. Geng J. Guo X. Ma J. Wang H. (2019). Intracellular delivery of nucleic acid by cell-permeable hPP10 peptide. J. Cell Physiol. 234 (7), 11670–11678. 10.1002/jcp.27826 30515802

[B30] Duan Z. Duan Y. Lamendola D. E. Yusuf R. Z. Naeem R. Penson R. T. (2003). Overexpression of MAGE/GAGE genes in paclitaxel/doxorubicin-resistant human cancer cell lines. Clin. Cancer Res. 9 (7), 2778–2785. 12855658

[B31] Duan Z. Feller A. J. Toh H. C. Makastorsis T. Seiden M. V. (1999). TRAG-3, a novel gene, isolated from a taxol-resistant ovarian carcinoma cell line. Gene 229 (1), 22975–22981. 10.1016/s0378-1119(99)00042-6 10095106

[B32] Edelman R. (2002). The development and use of vaccine adjuvants, applied biochemistry and Biotechnology - Part B molecular Biotechnology. Mol. Biotechnol. 21, 129–148. 10.1385/mb:21:2:129 12059113

[B34] Esfandiary A. Ghafouri-Fard S. (2015). MAGE-A3: An immunogenic target used in clinical practice. Immunotherapy 7, 683–704. 10.2217/imt.15.29 26100270

[B35] Fan R. Huang W. Luo B. Zhang Q. M. Xiao S. W. Xie X. X. (2015). Cancer testis antigen OY-TES-1: Analysis of protein expression in ovarian cancer with tissue microarrays. Eur. J. Gynaecol. Oncol. 36 (3), 298–303. 26189257

[B36] Felder M. Kapur A. Gonzalez-Bosquet J. Horibata S. Heintz J. Albrecht R. (2014). MUC16 (CA125): Tumor biomarker to cancer therapy, a work in progress. Mol. Cancer 1313, 129–144. Molecular Cancer. BioMed Central Ltd.;. 10.1186/1476-4598-13-129 PMC4046138 24886523

[B37] Feni L. Neundorf I. (2017). The current role of cell-penetrating peptides in cancer therapy. Adv. Exp. Med. Biol. 1030, 279–295. 10.1007/978-3-319-66095-0_13 29081059

[B38] Foged C. Brodin B. Frokjaer S. Sundblad A. (2005). Particle size and surface charge affect particle uptake by human dendritic cells in an *in vitro* model. Int. J. Pharm. 298, 315–322. 10.1016/j.ijpharm.2005.03.035 15961266

[B39] Franzese E. Centonze S. Diana A. Carlino F. Guerrera L. P. Di Napoli M. (2019). PARP inhibitors in ovarian cancer. Cancer Treat. Rev. 73, 1–9. 10.1016/j.ctrv.2018.12.002 30543930

[B40] Fukuhara H. Ino Y. Todo T. (2016). Oncolytic virus therapy: A new era of cancer treatment at dawn. Cancer Sci. 107, 1373–1379. 10.1111/cas.13027 27486853 PMC5084676

[B41] Gao Q. Xiang S. D. Wilson K. Madondo M. Stephens A. N. Plebanski M. (2018). Sperm protein 17 expression by murine epithelial ovarian cancer cells and its impact on tumor progression. Cancers (Basel) 10 (8). 10.3390/cancers10080276 PMC6115966 30127274

[B42] Garg M. Chaurasiya D. Rana R. Jagadish N. Kanojia D. Dudha N. (2007). Sperm-associated antigen 9, a novel cancer testis antigen, is a potential target for immunotherapy in epithelial ovarian cancer. Clin. Cancer Res. 13 (5), 1421–1428. 10.1158/1078-0432.ccr-06-2340 17332284

[B43] Gillespie A. M. Rodgers S. Wilson A. P. Tidy J. Rees R. C. Coleman R. E. (1998). MAGE, BAGE and GAGE: Tumour antigen expression in benign and malignant ovarian tissue. Br. J. Cancer 78 (6), 816–821. 10.1038/bjc.1998.585 9743307 PMC2062964

[B44] Godefroy E. Wang Y. Souleimanian N. E. Scotto L. Stevanovic S. Chen Y. T. (2007). Assessment of CD4+ T cells specific for the tumor antigen SSX-1 in cancer-free individuals. Cancer Immunol. Immunother. 56 (8), 1183–1192. 10.1007/s00262-006-0269-9 17186289 PMC11030208

[B45] Gray H. J. Benigno B. Berek J. Chang J. Mason J. Mileshkin L. (2016). Progression-free and overall survival in ovarian cancer patients treated with CVac, a mucin 1 dendritic cell therapy in a randomized phase 2 trial. J. Immunother. Cancer 4 (34), 27330807. 10.1186/s40425-016-0137-x PMC4915201 27330807

[B46] Griffioen M. Kessler J. H. Borghi M. van Soest R. A. van der Minne C. E. Nouta J. (2006). Detection and functional analysis of CD8+ T cells specific for PRAME: A target for T-cell therapy. Clin. Cancer Res. 12 (10), 3130–3136. 10.1158/1078-0432.ccr-05-2578 16707612

[B47] Gupta N. Jagadish N. Surolia A. Suri A. (2017). Heat shock protein 70-2 (HSP70-2) a novel cancer testis antigen that promotes growth of ovarian cancer. Am. J. Cancer Res. 7 (6), 1252–1269. 28670489 PMC5489776

[B48] Habault J. Poyet J. L. (2019). Recent advances in cell penetrating peptide-based anticancer therapies. Molecules 24 (5), 927. 10.3390/molecules24050927 PMC6429072 30866424

[B49] Hamanishi J. Mandai M. Ikeda T. Minami M. Kawaguchi A. Murayama T. (2015). Safety and antitumor activity of Anti-PD-1 antibody, nivolumab, in patients with platinum-resistant ovarian cancer. Jco 33 (34), 4015–4022. 10.1200/jco.2015.62.3397 26351349

[B50] Hasegawa K. Ono T. Matsushita H. Shimono M. Noguchi Y. Mizutani Y. (2004). A-kinase anchoring protein 3 messenger RNA expression in ovarian cancer and its implication on prognosis. Int. J. Cancer 108 (1), 86–90. 10.1002/ijc.11565 14618620

[B51] Heery C. R. Ibrahim N. K. Arlen P. M. Mohebtash M. Murray J. L. Koenig K. (2015). Docetaxel alone or in combination with a therapeutic cancer vaccine (PANVAC) in patients with metastatic breast cancer: A randomized clinical trial. JAMA Oncol. 1 (8), 1087–1095. 10.1001/jamaoncol.2015.2736 26291768 PMC6622177

[B52] Hoebe K. Janssen E. Beutler B. (2004). The interface between innate and adaptive immunity. Nat. Immunol. 5 (10), 971–974. 10.1038/ni1004-971 15454919

[B53] Hung C. Cheng W. Chai C. (2022). Improving vaccine potency through intercellular spreading and enhanced MHC class I presentation of antigen. J. Immunol. 166 (9), 5733–5740. 10.4049/jimmunol.166.9.5733 11313416

[B54] Hylander B. Repasky E. Shrikant P. Intengan M. Beck A. Driscoll D. (2006). Expression of Wilms tumor gene (WT1) in epithelial ovarian cancer. Gynecol. Oncol. 101 (1), 12–17. 10.1016/j.ygyno.2005.09.052 16263157

[B55] Kalli K. R. Block M. S. Kasi P. M. Erskine C. L. Hobday T. J. Dietz A. (2018). Folate receptor alpha peptide vaccine generates immunity in breast and ovarian cancer patients. Clin. Cancer Res. 24 (13), 3014–3025. 10.1158/1078-0432.ccr-17-2499 29545464 PMC6030477

[B56] Kandalaft L. E. Chiang C. L. Tanyi J. Motz G. Balint K. Mick R. (2013). A Phase I vaccine trial using dendritic cells pulsed with autologous oxidized lysate for recurrent ovarian cancer. J. Transl. Med. 11, 149. 10.1186/1479-5876-11-149 23777306 PMC3693890

[B57] Kawai T. Akira S. (2010). The role of pattern-recognition receptors in innate immunity: Update on toll-like receptors. Nat. Immunol. 11, 373–384. 10.1038/ni.1863 20404851

[B58] Kloudová K. Hromádková H. Partlová S. Brtnickỳ T. Rob L. Bartunková J. (2016). Expression of tumor antigens on primary ovarian cancer cells compared to established ovarian cancer cell lines. Oncotarget 7 (29), 46120–46126. 10.18632/oncotarget.10028 27323861 PMC5216785

[B59] Köbel M. Madore J. Ramus S. J. Clarke B. A. Pharoah D. P. Deen S. (2014). Evidence for a time-dependent association between FOLR1 expression and survival from ovarian carcinoma: Implications for clinical testing. An ovarian tumour tissue analysis consortium study. Br. J. Cancer 111, 2297–2307. 10.1038/bjc.2014.567 25349970 PMC4264456

[B60] Kreuzinger C. Geroldinger A. Smeets D. Braicu E. I. Sehouli J. Koller J. (2017). A complex network of tumor microenvironment in human high-grade serous ovarian cancer. Clin. Cancer Res. 23 (24), 7621–7632. 10.1158/1078-0432.ccr-17-1159 28972047

[B61] Kumar V. Jagadish N. Suri A. (2017). Role of A-Kinase anchor protein (AKAP4) in growth and survival of ovarian cancer cells. Oncotarget 8 (32), 53124–53136. 10.18632/oncotarget.18163 28881798 PMC5581097

[B62] Kurman R. J. Shih I-M. (2011). Molecular pathogenesis and extraovarian origin of epithelial ovarian cancer-Shifting the paradigm. Hum. Pathol. 42 (7), 918–931. 10.1016/j.humpath.2011.03.003 21683865 PMC3148026

[B63] Kurman R. J. Shih I-M. (2010). The origin and pathogenesis of epithelial ovarian cancer: A proposed unifying theory. Am. J. Surg. Pathol. 34 (3), 433–443. 10.1097/pas.0b013e3181cf3d79 20154587 PMC2841791

[B64] Kushwah R. Hu J. (2011). Complexity of dendritic cell subsets and their function in the host immune system. Immunology 133 (4), 409–419. 10.1111/j.1365-2567.2011.03457.x 21627652 PMC3143352

[B65] Lee E. K. Konstantinopoulos P. A. (2019). Combined PARP and immune checkpoint inhibition in ovarian cancer. Trends Cancer 5, 524–528. Cell Press. 10.1016/j.trecan.2019.06.004 31474356

[B66] Lee J. W. Foote R. S. (2009). Micro and nano technologies in Bioanalysis. Preface. Methods Mol. Biol. 544, 547–557. 10.1007/978-1-59745-483-4 19771672

[B67] Leung F. Dimitromanolakis A. Kobayashi H. Diamandis E. P. Kulasingam V. (2013). Folate-receptor 1 (FOLR1) protein is elevated in the serum of ovarian cancer patients. Clin. Biochem. 46 (15), 1462–1468. 10.1016/j.clinbiochem.2013.03.010 23528302 PMC4130215

[B68] Link P. A. Zhang W. Odunsi K. Karpf A. R. (2013). BORIS/CTCFL mRNA isoform expression and epigenetic regulation in epithelial ovarian cancer. Cancer Immun. 13 (1), 6. 23390377 PMC3559194

[B69] Lu L. Katsaros D. Wiley A. Rigault de la Longrais I. A. Puopolo M. Yu H. (2007). Expression of MDR1 in epithelial ovarian cancer and its association with disease progression. Oncol. Res. 16 (8), 395–403. 10.3727/000000006783980892 17913048

[B70] Ma J. Ren S. Ding J. Liu S. Zhu J. Ma R. (2019). Expression of RRBP1 in epithelial ovarian cancer and its clinical significance. Biosci. Rep. 39 (7). 10.1042/BSR20190656 PMC6646231 31285390

[B71] Materna V. Surowiak P. Kaplenko I. Spaczyński M. Duan Z. Zabel M. (2007). Taxol-resistance-associated gene-3 (TRAG-3/CSAG2) expression is predictive for clinical outcome in ovarian carcinoma patients. Virchows Arch. 450 (2), 187–194. 10.1007/s00428-006-0346-7 17216190

[B72] Mccarthy H. O. McCaffrey J. Mccrudden C. M. Zholobenko A. Ali A. A. McBride J. W. (2014). Development and characterization of self-assembling nanoparticles using a bio-inspired amphipathic peptide for gene delivery. J. Control Release 189, 141–149. 10.1016/j.jconrel.2014.06.048 24995949

[B73] McCluggage W. G. Hirschowitz F. R. C. Gilks L. Wilkinson F. R. C. Singh C. B. Wilkinson N. (2017). The fallopian tube origin and primary site assignment in extrauterine high-grade serous carcinoma: Findings of a survey of pathologists and clinicians. Int. J. Gynecol. Pathol. 36, 230–239. 10.1097/PGP.0000000000000336 27801755

[B74] McCormack E. Adams K. J. Hassan N. J. Kotian A. Lissin N. M. Sami M. (2013). Bi-specific TCR-anti CD3 redirected T-cell targeting of NY-ESO-1- and LAGE-1-positive tumors. Cancer Immunol. Immunother. 62 (4), 773–785. 10.1007/s00262-012-1384-4 23263452 PMC3624013

[B75] Mccrudden C. M. Mcbride J. W. Mccaffrey J. Mcerlean E. M. Dunne N. J. Kett V. L. (2018). Gene therapy with RALA/iNOS composite nanoparticles significantly enhances survival in a model of metastatic prostate cancer. Cancer Nanotechnol. 9 (1), 5. 10.1186/s12645-018-0040-x 29899810 PMC5982451

[B76] Mcerlean E. M. Mccrudden C. M. Mccarthy H. O. (2015). “Multifunctional delivery systems for cancer gene therapy,” in Gene therapy: Principles and challenges. Editor Doaa H. (Rijeka, Croatia: inTech). 10.5772/61297

[B77] Mittendorf E. A. Clifton G. T. Holmes J. P. Schneble E. van Echo D. Ponniah S. (2014). Final report of the phase I/II clinical trial of the E75 (nelipepimut-S) vaccine with booster inoculations to prevent disease recurrence in high-risk breast cancer patients. Ann. Oncol. 25 (9), 1735–1742. 10.1093/annonc/mdu211 24907636 PMC4143091

[B78] Mittendorf E. A. Holmes J. P. Ponniah S. Peoples G. E. (2008). The E75 HER2/neu peptide vaccine. Cancer Immunol. Immunother. 57, 1511–1521. Cancer Immunology, Immunotherapy. Springer. 10.1007/s00262-008-0540-3 18536917 PMC11029853

[B79] Mohebtash M. Tsang K. Y. Madan R. A. Huen N. Y. Poole D. J. Jochems C. (2011). A pilot study of MUC-1/CEA/TRICOM poxviral-based vaccine in patients with metastatic breast and ovarian cancer. Clin. Cancer Res. 17 (22), 7164–7173. 10.1158/1078-0432.ccr-11-0649 22068656 PMC3227395

[B80] Muhammad A. Champeimont J. Mayr U. B. Lubitz W. Kudela P. (2012). Bacterial ghosts as carriers of protein subunit and DNA-encoded antigens for vaccine applications. Expert Rev. Vaccines 11, 97–116. 10.1586/erv.11.149 22149712

[B81] Myc L. A. Gamian A. Myc A. (2011). Cancer vaccines . Any future. Arch. Immunol. Ther. Exp. Warsz. 59 (4), 249–259. 10.1007/s00005-011-0129-y 21644030

[B82] Neek M. Kim T. I. Wang S. (2019). Protein-based nanoparticles in cancer vaccine development. Nanomedicine Nanotechnol. Biol. Med. 15 (1), 164–174. 10.1016/j.nano.2018.09.004 PMC6289732 30291897

[B83] Neff R. T. Senter L. Salani R. (2017). BRCA mutation in ovarian cancer: Testing, implications and treatment considerations. Ther. Adv. Med. Oncol. 9 (8), 519–531. 10.1177/1758834017714993 28794804 PMC5524247

[B84] Niu X. Gao Z. Qi S. Su L. Yang N. Luan X. (2018). Macropinocytosis activated by oncogenic Dbl enables specific targeted delivery of Tat/pDNA nano-complexes into ovarian cancer cells. Int. J. Nanomedicine 13, 4895–4911. 10.2147/ijn.s171361 30214196 PMC6122892

[B85] Odunsi K. (2017). Immunotherapy in ovarian cancer. Ann. Oncol. 288, viii1–7. 10.1093/annonc/mdx444 PMC5834124 29232467

[B86] Odunsi K. (2017). Immunotherapy in ovarian cancer. Ann. Oncol. 28 (8), viii1–788. Paradoxical Role of Trpv1.Pdf. 10.1093/annonc/mdx444 29232467 PMC5834124

[B87] Odunsi K. Jungbluth A. A. Stockert E. Qian F. Gnjatic S. Tammela J. (2003). NY-ESO-1 and LAGE-1 cancer-testis antigens are potential targets for immunotherapy in epithelial ovarian cancer. Cancer Res. 63 (18), 6076–6083. 14522938

[B88] Pankov D. Sjöström L. Kalidindi T. Lee S. G. Sjöström K. Gardner R. (2017). *In vivo* immuno-targeting of an extracellular epitope of membrane bound preferentially expressed antigen in melanoma (PRAME). Oncotarget 8 (39), 65917–65931. 10.18632/oncotarget.19579 29029482 PMC5630382

[B89] Perales-Puchalt A. Wojtak K. Duperret E. K. Yang X. Slager A. M. Yan J. (2019). Engineered DNA vaccination against follicle-stimulating hormone receptor delays ovarian cancer progression in animal models. Mol. Ther. 27, 314–325. 10.1016/j.ymthe.2018.11.014 30554854 PMC6369450

[B90] Petrovsky N. (2006). Novel human polysaccharide adjuvants with dual Th1 and Th2 potentiating activity. Vaccine 24 (2), S26. 10.1016/j.vaccine.2005.01.107 PMC3101117 16823913

[B91] Piek J. M. J. van Diest P. J. Zweemer R. P. Jansen J. W. Poort-Keesom R. J. J. Poort-Keesom F. H. (2001). Dysplastic changes in prophylactically removed Fallopian tubes of women predisposed to developing ovarian cancer. J. Pathol. 195 (4), 451–456. 10.1002/path.1000 11745677

[B92] Rahman F. Dahmen A. Herzog-Hauff S. Böcher W. O. Galle P. R. Löhr H. E. (2000). Cellular and humoral immune responses induced by intradermal or intramuscular vaccination with the major Hepatitis B surface antigen. Hepatology 31, 521–527. 10.1002/hep.510310237 10655280

[B93] Restifo N. P. Ying H. Hwang L. Leitner W. W. (2000). The promise of nucleic acid vaccines. Gene Ther. 7 (2), 89–92. 10.1038/sj.gt.3301117 10673713 PMC2241736

[B94] Sabbatini P. Tsuji T. Ferran L. Ritter E. Sedrak C. Tuballes K. (2012). Phase I trial of overlapping long peptides from a tumor self-antigen and poly-ICLC shows rapid induction of integrated immune response in ovarian cancer patients. Clin. Cancer Res. 18 (23), 6497–6508. 10.1158/1078-0432.ccr-12-2189 23032745

[B95] Sallum L. F. Andrade L. Ramalho S. Ferracini A. C. de Andrade Natal R. de A. Brito A. B. (2018). WT1, p53 and p16 expression in the diagnosis of low- and high-grade serous ovarian carcinomas and their relation to prognosis. Oncotarget 9 (22), 15818–15827. 10.18632/oncotarget.24530 29662608 PMC5882299

[B96] Shahzad M. M. K. Shin Y. H. Matsuo K. Lu C. Nishimura M. Shen D. Y. (2013). Biological significance of HORMA domain containing protein 1 (HORMAD1) in epithelial ovarian carcinoma. Cancer Lett. 330, 123–129. Elsevier Ireland Ltd. 10.1016/j.canlet.2012.07.001 22776561 PMC3498611

[B97] Sharma A. Albahrani M. Zhang W. Kufel C. N. James S. R. Odunsi K. (2019). Epigenetic activation of POTE genes in ovarian cancer. Epigenetics 14 (2), 185–197. 10.1080/15592294.2019.1581590 30764732 PMC6557602

[B98] Sharma S. Qian F. Keitz B. Driscoll D. Scanlan M. J. Skipper J. (2005). A-kinase anchoring protein 3 messenger RNA expression correlates with poor prognosis in epithelial ovarian cancer. Gynecol. Oncol. 99 (1), 183–188. 10.1016/j.ygyno.2005.06.006 16005946

[B99] Sharma S. Qian F. Keitz B. Driscoll D. Scanlan M. J. Skipper J. (2005). A-kinase anchoring protein 3 messenger RNA expression correlates with poor prognosis in epithelial ovarian cancer. Gynecol. Oncol. 99, 183–188. Gynecologic Oncology. Academic Press. 10.1016/j.ygyno.2005.06.006 16005946

[B100] Sheng N. Xu Y-Z. Xi Q-H. Jiang H-Y. Wang C-Y. Zhang Y. (2018). Overexpression of KIF2A is suppressed by miR-206 and associated with poor prognosis in ovarian cancer. Cell Physiol. Biochem. 50 (3), 810–822. 10.1159/000494467 30352438

[B101] Silwal-Pandit L. Langerød A. Børresen-Dale A. L. (2017). TP53Mutations in breast and ovarian cancer. Cold Spring Harb. Perspect. Med. 7 (1), a026252. 10.1101/cshperspect.a026252 27815305 PMC5204332

[B102] Smith H. A. McNeel D. G. (2010). The SSX family of cancer-testis antigens as target proteins for tumor therapy. Clin. Dev. Immunol. 2010, 150591. 10.1155/2010/150591 20981248 PMC2963798

[B103] Sokołowska E. Błachnio-Zabielska A. U. (2019). A critical review of electroporation as A plasmid delivery system in mouse skeletal muscle. Int. J. Mol. Sci. 20 (11), 2776. 10.3390/ijms20112776 PMC6600476 31174257

[B104] Speiser D. E. Lí Enard D. El M. Pittet J. Batard P. Rimoldi D. (2002). *In vivo* activation of melanoma-specific CD8 + T cells by endogenous tumor antigen and peptide vaccines. A comparison to virus-specific T cells. Eur. J. Immunol. 32 (3), 731–741. 10.1002/1521-4141(200203)32:3<731:AID-IMMU731>3.0.CO;2-H 11870617

[B105] Srdelić S. Kuzmić-Prusac I. Spagnoli G. C. Juretić A. Čapkun V. (2019). MAGE-A4 and MAGE-A1 immunohistochemical expression in high-grade endometrial cancer. Int. J. Gynecol. Pathol. 38 (1), 59–65. 10.1097/pgp.0000000000000470 29140883

[B106] Stewart C. J. R. Brennan B. A. Chan T. Netreba J. (2008). WT1 expression in endometrioid ovarian carcinoma with and without associated endometriosis. Pathology 40 (6), 592–599. 10.1080/00313020802320697 18752126

[B107] Straughn J. M. Shaw D. R. Guerrero A. Bhoola S. M. Racelis A. Wang Z. (2004). Expression of sperm protein 17 (Sp17) in ovarian cancer. Int. J. Cancer 108 (6), 805–811. 10.1002/ijc.11617 14712480

[B108] Stuart G. C. E. (2003). First-line treatment regimens and the role of consolidation therapy in advanced ovarian cancer. Gynecol. Oncol. 90, S8–S15. Gynecologic Oncology. Academic Press Inc. 10.1016/s0090-8258(03)00472-4 13129490

[B109] Takeuchi O. Akira S. (2010). Pattern recognition receptors and inflammation. Cell 140 (6), 805–820. 10.1016/j.cell.2010.01.022 20303872

[B110] Tanyi J. L. Bobisse S. Ophir E. Tuyaerts S. Roberti A. Genolet R. (2018). Personalized cancer vaccine effectively mobilizes antitumor T cell immunity in ovarian cancer. Sci. Transl. Med. 10 (436). 10.1126/scitranslmed.aao5931 29643231

[B111] Tawde S. A. Chablani L. Akalkotkar A. D'Souza M. J. (2016). Evaluation of microparticulate ovarian cancer vaccine via transdermal route of delivery. J. Control. Release 235, 147–154. 10.1016/j.jconrel.2016.05.058 27238440

[B112] Temizoz B. Kuroda E. Ishii K. J. (2016). Vaccine adjuvants as potential cancer immunotherapeutics. Intimm 28 (7), 329–338. 10.1093/intimm/dxw015 PMC4922024 27006304

[B113] Trnski D. Gregorić M. Levanat S. Ozretić P. Rinčić N. Vidaković T. (2019). Regulation of survivin isoform expression by GLI proteins in ovarian cancer. Cells 8 (2), 128. 10.3390/cells8020128 PMC6406444 30736319

[B114] Tsibulak I. Wieser V. Degasper C. Shivalingaiah G. Wenzel S. Sprung S. (2018). BRCA1 and BRCA2 mRNA-expression prove to be of clinical impact in ovarian cancer. Br. J. Cancer 119, 683–692. 10.1038/s41416-018-0217-4 30111871 PMC6173779

[B115] Türeci Ö. Chen Y. T. Sahin U. Güre A. O. Zwick C. Villena C. (1998). Expression of SSX genes in human tumors. Int. J. Cancer [Internet] 77 (1), 19–23. 10.1002/(sici)1097-0215(19980703)77:1<19::aid-ijc4>3.0.co;2-2 9639388

[B116] Turnis M. E. Rooney C. M. (2010). Enhancement of dendritic cells as vaccines for cancer. Immunotherapy 2 (6), 847–862. 10.2217/imt.10.56 21091116 PMC3433954

[B117] Udhayakumar V. K. Beuckelaer A. D. Mccaffrey J. Mccrudden C. M. Kirschman J. L. Vanover D. (2017). Arginine-rich peptide-based mRNA nanocomplexes efficiently instigate cytotoxic T cell immunity dependent on the amphipathic organization of the peptide. Adv. Healthc. Mat. 6 (13), 1–13. 10.1002/adhm.201601412 28436620

[B118] Vaidyanathan A. Sawers L. Gannon A. L. Chakravarty P. Scott A. L. Bray S. E. (2016). ABCB1 (MDR1) induction defines a common resistance mechanism in paclitaxel- and olaparib-resistant ovarian cancer cells. Br. J. Cancer 115 (4), 431–441. 10.1038/bjc.2016.203 27415012 PMC4985349

[B119] Valmori D. Qian F. Ayyoub M. Renner C. Merlo A. Gjnatic S. (2006). Expression of synovial sarcoma X (SSX) antigens in epithelial ovarian cancer and identification of SSX-4 epitopes recognized by CD4+ T cells. Clin. Cancer Res. 12 (2), 398–404. 10.1158/1078-0432.ccr-05-1902 16428478

[B120] van der Gun B. T. F. Huisman C. Stolzenburg S. Kazemier H. G. Ruiters M. H. J. Blancafort P. (2013). Bidirectional modulation of endogenous EpCAM expression to unravel its function in ovarian cancer. Br. J. Cancer 108 (4), 881–886. 10.1038/bjc.2013.45 23403823 PMC3590680

[B121] Van der Heijden I. Beijnen H. J. Nuijen B. (2013). Int. J. Pharm. Long term Stab. lyophilized plasmid DNA pDERMATT 453 (2), 648–650. 10.1016/j.ijpharm.2013.06.010 23792100

[B122] Van Elssen C. H. M. J. Frings P. W. H. Bot F. J. Van de Vijver K. K. Huls M. B. Meek B. (2010). Expression of aberrantly glycosylated Mucin-1 in ovarian cancer. Histopathology 57 (4), 597–606. 10.1111/j.1365-2559.2010.03667.x 20955385

[B123] Verri E. Guglielmini P. Puntoni M. Perdelli L. Papadia A. Lorenzi P. (2005). HER2/neu oncoprotein overexpression in epithelial ovarian cancer : Evaluation of its prevalence and prognostic significance. Clinical study. Oncology 68, 154–161. 10.1159/000086958 16020953

[B124] Verri E. Guglielmini P. Puntoni M. Perdelli L. Papadia A. Lorenzi P. (2020). HER2/neu oncoprotein overexpression in epithelial ovarian cancer: Evaluation of its prevalence and prognostic significance. Clinical study. Oncology 68 (2–3), 154–161. 10.1159/000086958 16020953

[B125] Wang B. Li X. Zhao G. Yan H. Dong P. Watari H. (2018). miR-203 inhibits ovarian tumor metastasis by targeting BIRC5 and attenuating the TGFβ pathway. J. Exp. Clin. Cancer Res. 37 (1), 235. 10.1186/s13046-018-0906-0 30241553 PMC6150978

[B126] Wang D. Zhu H. Ye Q. Wang C. Xu Y. (2016). Prognostic value of KIF2A and HER2-neu overexpression in patients with epithelial ovarian cancer. Med. Baltim. 95 (8), e2803. 10.1097/MD.0000000000002803 PMC4779007 26937910

[B127] Wang L. Mao Y. Du G. He C. Han S. (2015). Overexpression of JARID1B is associated with poor prognosis and chemotherapy resistance in epithelial ovarian cancer. Tumor Biol. 36 (4), 2465–2472. 10.1007/s13277-014-2859-z PMC4428534 25663457

[B128] Wang R. Wang H. Y. (2016). Immune targets and neoantigens for cancer immunotherapy and precision medicine. Cell Res. 27 (1), 11–37. 10.1038/cr.2016.155 28025978 PMC5223235

[B129] Wang T. Upponi J. R. Torchilin V. P. (2012). Review Design of multifunctional non-viral gene vectors to overcome physiological barriers: Dilemmas and strategies. Int. J. Pharm. 427, 3–20. 10.1016/j.ijpharm.2011.07.013 21798324

[B130] Wang Z. Zhang J. Zhang Y. Deng Q. Liang H. (2018). Expression and mutations of BRCA in breast cancer and ovarian cancer: Evidence from bioinformatics analyses. Int. J. Mol. Med. 42 (6), 3542–3550. 10.3892/ijmm.2018.3870 30221688

[B131] Woloszynska-Read A. James S. R. Link P. A. Yu J. Odunsi K. Karpf A. R. (2007). DNA methylation-dependent regulation of BORIS/CTCFL expression in ovarian cancer. Cancer Immun. 7, 21. 18095639 PMC2935752

[B132] Woloszynska-Read A. Zhang W. Yu J. Link P. A. Mhawech-Fauceglia P. Collamat G. (2011). Coordinated cancer germline antigen promoter and global DNA hypomethylation in ovarian cancer: Association with the BORIS/CTCF expression ratio and advanced stage. Clin. Cancer Res. 17 (8), 2170–2180. 10.1158/1078-0432.ccr-10-2315 21296871 PMC3079045

[B133] Wu D. di Li Li X. Meng X. N. Yan J. Zonghong Z. (2016). MicroRNA-873 mediates multidrug resistance in ovarian cancer cells by targeting ABCB1. Tumor Biol. 37 (8), 10499–10506. 10.1007/s13277-016-4944-y 26850595

[B134] Xie K. Fu C. Wang S. Xu H. Liu S. Shao Y. (2019). Cancer-testis antigens in ovarian cancer: Implication for biomarkers and therapeutic targets. J. Ovarian Res. 12, 1–13. 10.1186/s13048-018-0475-z 30609934 PMC6318940

[B135] Yakirevich E. Sabo E. Lavie O. Mazareb S. Spagnoli G. C. Resnick M. B. (2003). Expression of the MAGE-A4 and NY-ESO-1 cancer-testis antigens in serous ovarian neoplasms. Clin. Cancer Res. 9, 6453–6460. 14695148

[B136] Yao X. Hu J. F. Li T. Yang Y. Sun Z. Ulaner G. A. (2004). Epigenetic regulation of the taxol resistance-associated gene TRAG-3 in human tumors. Cancer Genet. Cytogenet. 151 (1), 1–13. 10.1016/j.cancergencyto.2003.08.021 15120907

[B137] Zhang S. Zhou X. Yu H. Yu Y. (2010). Expression of tumor-specific antigen MAGE, GAGE and BAGE in ovarian cancer tissues and cell lines. BMC Cancer 10, 163–169. 10.1186/1471-2407-10-163 20423514 PMC2868811

[B138] Zhang W. Barger C. J. Eng K. H. Klinkebiel D. Link P. A. Omilian A. (2016). PRAME expression and promoter hypomethylation in epithelial ovarian cancer. Oncotarget 7 (29), 45352–45369. 10.18632/oncotarget.9977 27322684 PMC5216727

[B139] Zhang W. Barger C. J. Link P. A. Mhawech-Fauceglia P. Miller A. Akers S. N. (2015). DNA hypomethylation-mediated activation ofCancer/Testis Antigen 45(CT45) genes is associated with disease progression and reduced survival in epithelial ovarian cancer. Epigenetics 10 (8), 736–748. 10.1080/15592294.2015.1062206 26098711 PMC4622579

[B140] Zhu D. Shi C. Jiang Y. Zhu K. Wang X. Feng W. (2021). Cisatracurium inhibits the growth and induces apoptosis of ovarian cancer cells by promoting lincRNA-p21. Bioengineered 12 (1), 1505–1516. 10.1080/21655979.2021.1916271 33944652 PMC8806207

